# Early brain-wide disruption of sleep microarchitecture in amyotrophic lateral sclerosis

**DOI:** 10.1172/JCI194555

**Published:** 2025-11-06

**Authors:** Christina Lang, Simon J. Guillot, Dorothee Lule, Luisa T. Balz, Antje Knehr, Patrick Weydt, Johannes Dorst, Katharina Kandler, Hans-Peter Muller, Jan Kassubek, Laura Wassermann, Sandrine Da Cruz, Francesco Roselli, Albert C. Ludolph, Matei Bolborea, Luc Dupuis

**Affiliations:** 1 Department of Neurology, University Hospital of Ulm, Ulm, Germany.; 2 Deutsches Zentrum für Neurodegenerative Erkrankungen (DZNE), Ulm, Germany.; 3 University of Strasbourg, INSERM, Strasbourg Translational Neuroscience and Psychiatry STEP – CRBS, UMR-S 1329, Strasbourg, France.; 4 DZNE, Bonn, Germany.; 5 VIB-KU Leuven Center for Brain and Disease Research and Department of Neurosciences, KU Leuven, Leuven, Belgium.

**Keywords:** Genetics, Neuroscience, ALS

## Abstract

**BACKGROUND:**

Amyotrophic lateral sclerosis (ALS), the major adult-onset motor neuron disease, is preceded by an early period unrelated to motor symptoms, including altered sleep, with increased wakefulness and decreased deep nonrapid eye movement (NREM). Whether these alterations in sleep macroarchitecture are associated with — or even precede — abnormalities in sleep-related EEG features remains unknown.

**METHODS:**

Here, we characterize sleep microarchitecture using polysomnography for patients with ALS (*n* = 33) and controls (*n* = 32) as well as for asymptomatic carriers of superoxide dismutase 1 (*SOD1*) or *C9ORF72* mutations (*n* = 57) and noncarrier controls (*n* = 30). Patients and controls with factors that could confound sleep structure, including respiratory insufficiency, were prospectively excluded. The results were complemented in 3 ALS mouse models (*Sod1*^G86R^, *Fus*^ΔNLS/+^, and *TDP-43*^Q331K^).

**RESULTS:**

We observed a brain-wide reduction in the density of sleep spindles, slow oscillations, and K-complexes in patients with early-stage ALS and in presymptomatic gene carriers. These defects in sleep spindles and slow oscillations correlated with cognitive performance in both cohorts, particularly with scores on memory, verbal fluency, and language function. Alterations in sleep microarchitecture were replicated in 3 mouse models, and decreases in sleep spindles were rescued following intracerebroventricular supplementation of melanin-concentrating hormone (MCH) or by oral administration of a dual orexin receptor antagonist.

**CONCLUSION:**

Sleep microarchitecture was associated with cognitive deficits and causally linked to aberrant MCH and orexin signaling in ALS.

**FUNDING:**

Agence Nationale de la Recherche (ANR); Fondation Thierry Latran; Association Francaise de Recherche sur la sclérose latérale amyotrophique; Association Française contre les myopathies; TargetALS; and Joint Program on Neurodegenerative Diseases Research (JPND).

## Abstract 2

We show that disruption in EEG patterns related to sleep, in particular sleep spindles and slow oscillations, are predating ALS onset for more than 10 years, and identify in mouse models possible therapeutic strategies to rescue this phenotype.

## Introduction

Amyotrophic lateral sclerosis (ALS) is a fatal and rapidly progressive disease affecting upper and lower motor neurons in adults, with a median survival of 3–4 years after the onset of motor symptoms. ALS usually manifests between 60 and 70 years of age (1, 2), and most patients lack a family history of the disease. However, 5%–10% of ALS cases are familial, with more than 40 distinct genes currently associated and mutations in *C9ORF72*, superoxide dismutase 1 (*SOD1*), TAR-DNA-binding protein (*TARDBP*), and fused in sarcoma (*FUS*) recognized as major genetic causes of ALS (1, 2).

It is generally considered that patients with ALS do not exhibit clinical manifestations before the onset of motor symptoms. Circulating neurofilament levels, a reliable biomarker of (motor) axonal injury, increase at the time of motor symptom onset, but not 1–2 years prior to it (3, 4). Clinically, most presymptomatic gene carriers do not show even mild motor impairment within this timeframe (4, 5). However, a number of early nonmotor signs, such as weight loss (6–10) or cognitive impairment (11, 12), are known to be present in the prodromal phase, many years before the appearance of motor symptoms.

In recent research, we identified sleep alterations as a novel early nonmotor sign of ALS. We investigated sleep in 2 cohorts of patients with ALS devoid of respiratory insufficiency and in presymptomatic gene carriers. In both cohorts, we observed significant defects in sleep macroarchitecture characterized by increased wakefulness and reduced deep sleep (nonrapid eye movement 2/3 [NREM2/3]) (13). These defects were detectable at least 10–15 years before the expected onset of motor symptoms in presymptomatic gene carriers and correlated with cognitive scores (13). Similar macroarchitectural alterations were observed in mouse models of familial ALS (13).

While previous studies established sleep defects as an early phenotype in individuals at high risk of developing ALS, we now sought to explore how sleep-related EEG hallmarks are affected in ALS that could be used in regular clinical assessment. The different sleep stages are distinguished by specific graphoelements in the EEG that reflect the activation of particular cortical and subcortical pathways. These neuronal activity patterns are considered manifestations of the microarchitecture of sleep. Key EEG hallmarks include sleep spindles, bursts of 11–15 Hz (sigma frequency band) activity, typically between 0.5 and 2 seconds in duration (14, 15), as well as slow oscillations (<1.5 Hz) and K-complexes (<1 Hz), all of which are involved in NREM sleep continuity and the role of sleep in memory consolidation and cognitive function (14, 16). A disruption in the microarchitecture of sleep could be caused by specific changes in neuroanatomical pathways and thus also serve as a potential biomarker for neuronal changes or pathological conversion. Here, we analyzed sleep microarchitecture components in patients with ALS, individuals who are presymptomatic gene carriers, and mouse models (13). We observed a widespread reduction in sleep spindles, slow oscillations, and K-complexes that correlated with cognitive function. We also observed similar changes in mouse models and demonstrated that they can be corrected by melanin-concentrating hormone (MCH) supplementation or the administration of a dual orexin receptor antagonist. Thus, sleep microarchitecture dynamics are affected early in ALS and linked to hypothalamic dysfunction.

## Results

**Figure 1:**
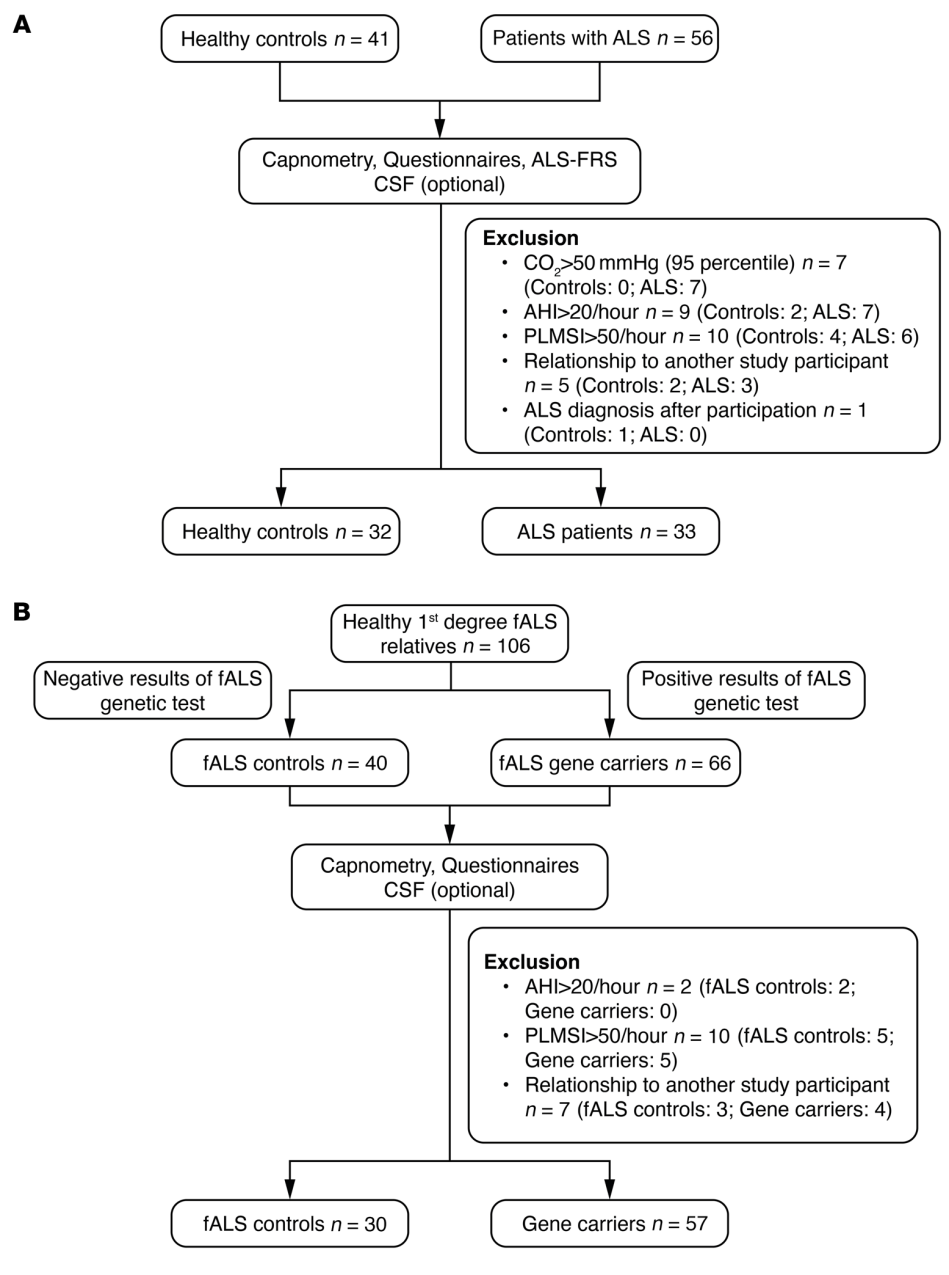
Flowchart of the 2 cohort studies. (**A** and **B**) Flowchart of the studies involving patients with ALS (**A**, same cohort as in ref. 13) and presymptomatic gene carriers (including additional individuals as in ref. 13).

Patients with early-stage ALS exhibit brain-wide decreased sleep spindle density. To examine the extent of alterations in sleep microarchitecture in individuals with early-stage ALS, we took advantage of polysomnography data acquired in a previous cohort study (13). The characteristics of the patients included in the study are described in Figure 1A and detailed in Table 1. We prospectively excluded patients and controls with abnormal capnographies, ensuring that the observed sleep defects were not secondary to respiratory insufficiency. Given the high prevalence of periodic limb movements in neurological diseases and the higher number of individuals with sleep-disordered breathing among patients with ALS, we determined the following exclusion criteria: apnea-hypopnea index (AHI) of higher than 20 and periodic limb movement index (PLMI) of higher than 50, reasoning that individuals above these thresholds would very likely display altered sleep architecture unrelated to ALS. We also did not observe REM behavior disorder or REM sleep without atonia in the patients or individuals included using established criteria (17). Furthermore, other potential factors contributing to alterations in sleep architecture, including sleep-related breathing disorders and periodic limb movements during sleep, were considered exclusion criteria. No significant differences were observed between the ALS patients and the control group with regard to age, sex or body mass index. Two patients with ALS (6%) were taking sleep-inducing medication (1 who was taking 2 mg melatonin, and 1 taking 22.5 mg mirtazapine), while no participants in the healthy control group or the presymptomatic gene carrier cohort were taking such medication.

**Figure 2:**
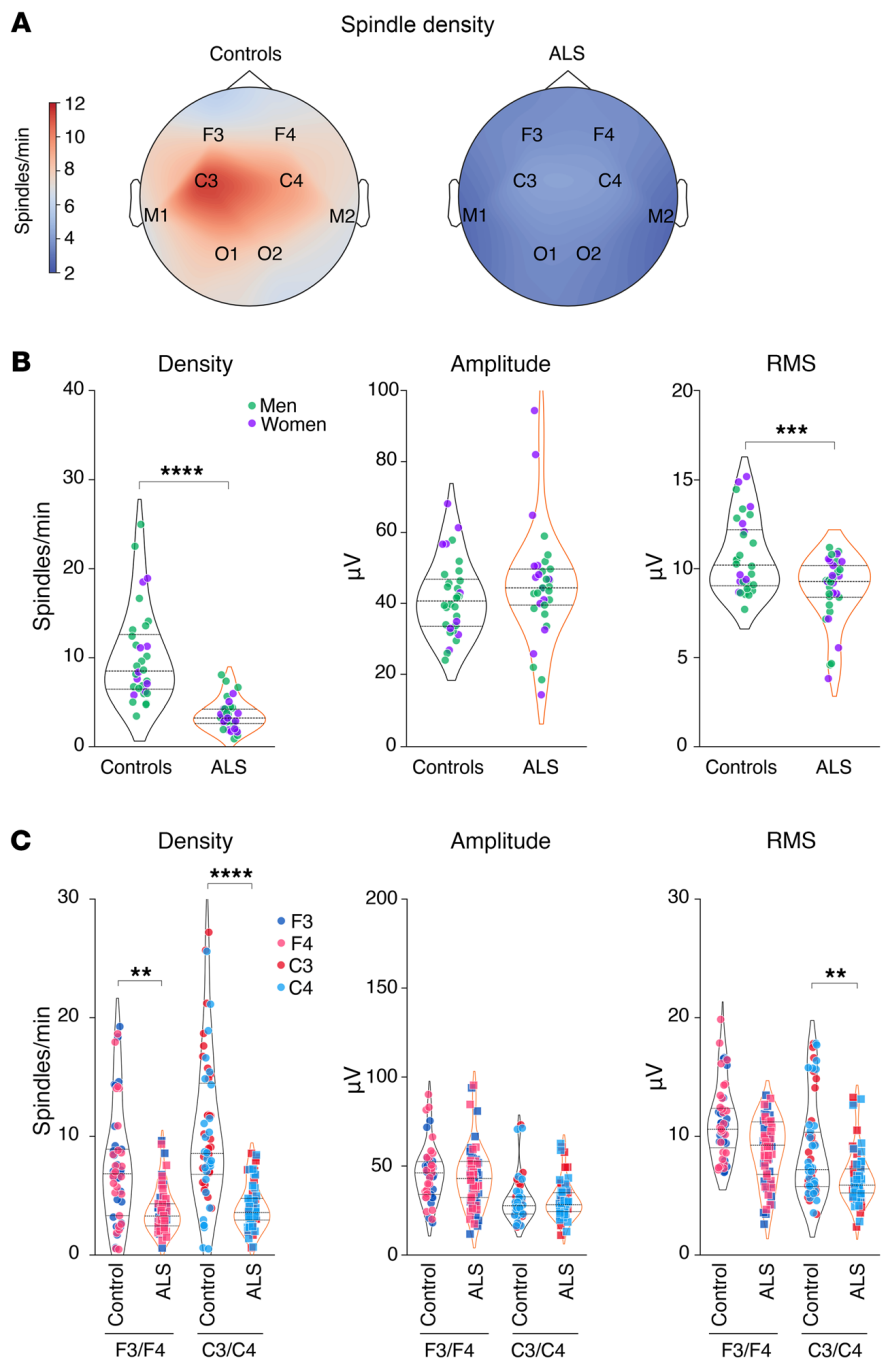
Sleep spindle alterations in patients with early-stage ALS. (**A**) Topographic map across all electrodes of sleep spindle density in controls and patients with ALS. (**B**) Quantification of sleep spindle density, amplitude, and RMS. ***P_adj._ < 0.001 and ****P_adj._< 0.0001, by independent 2-tailed Student’s *t* test with Welch’s *t* test correction. (**C**) Quantification of sleep spindle density, amplitude, and RMS across F3/F4 and C3/C4 electrodes. **P_adj._ < 0.01 and ****P_adj._< 0.0001, by Kruskal-Wallis test with Dunn’s multiple tests adjusted with FDR-BKY correction. Results with *P* > 0.05 are not indicated. Data are presented as the median and IQRs. Corrected *P* values are shown.

**Figure 3:**
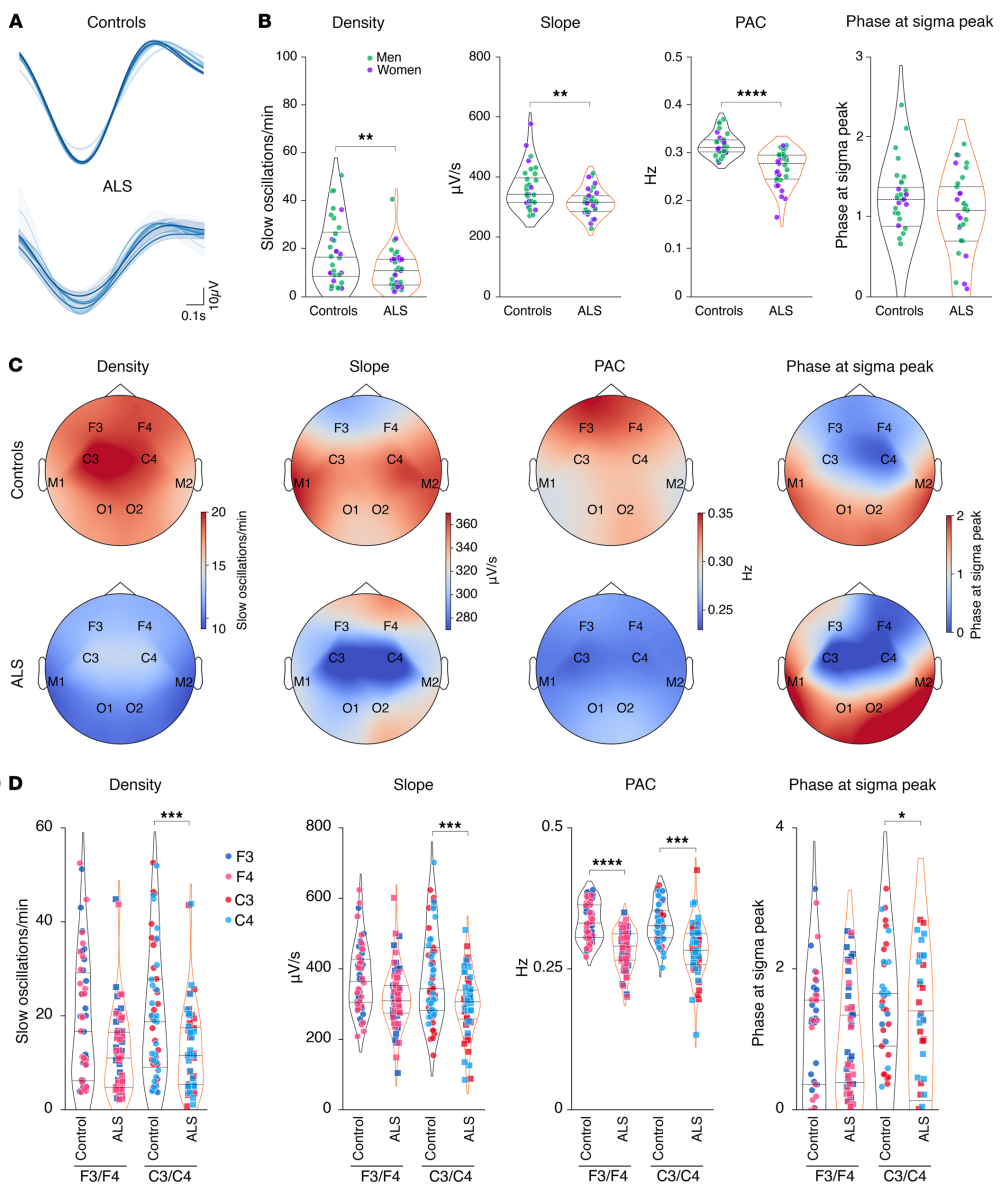
Slow oscillation alterations in patients with early-stage ALS. (**A**) Representative slow oscillations across all electrodes on a healthy individual and 1 patient with sALS. (**B**) Quantification of slow oscillation density, slope, and PAC between sleep spindles and slow oscillations (PAC) and phase at sigma peak (PSP) in controls and patients with ALS. **P_adj._ < 0.01 and ****P_adj._ < 0.0001, by independent 2-tailed Student’s *t* test with Welch’s *t* test correction. (**C**) Topographic maps across all electrodes of slow oscillation density, slope, PAC, and PSP in controls and patients with ALS. (**D**) Quantification of slow oscillation density, slope, PAC, and PSP across F3/F4 and C3/C4 electrodes as indicated. *P_adj._ < 0.05, ***P_adj._ < 0.001, and ****P_adj._ < 0.0001, by Kruskal-Wallis test with Dunn’s multiple tests adjusted with FDR-BKY correction. Results with *P* > 0.05 are not indicated. Data are presented as medians and IQRs. Corrected *P* values are shown.

Consistent with the previously observed strong defect in NREM2 and -3, we observed a pronounced decrease in sleep spindle density and their root mean square (RMS) in patients with ALS (Figure 2, A and B), while their amplitude was unchanged (Figure 2B). Topographically, we observed a significant decrease in sleep spindle density in both frontal and central electrodes (Figure 2C), while the RMS of sleep spindles was only significantly decreased in central electrodes (C3/C4) but not in frontal cortex electrodes (F3/F4) (Figure 2C). Parallel to the decreased sleep spindle density, we observed similar brain-wide reductions in slow oscillations (Figure 3, A–D) and K-complexes in individuals with early-stage ALS (Supplemental Figure 1; supplemental material available online with this article; https://doi.org/10.1172/JCI194555DS1). These results were not modified if individuals with intermediate AHI (5< AHI<20) and/or PMLSI (25<PMLSI<50) values were excluded from the analysis (Supplemental Figure 2). These observations indicate that sleep microarchitecture -s disrupted across the entire brain in patients with early-stage ALS.

### Presymptomatic ALS gene carriers show decreased sleep spindle density.

To further characterize the changes in the microarchitecture of sleep and to assess their onset during the course of the disease, a second prospective cohort study was conducted comprising presymptomatic ALS gene carriers, using identical inclusion and exclusion criteria (Figure 1B). This cohort corresponds to the cohort previously described (13), with an additional 29 gene carriers and 11 first-degree relatives of patients with familial ALS (fALS) with negative genetic reports. A total of 57 presymptomatic gene carriers (*SOD1* *n* = 13; *C9ORF72* *n* = 33; remaining individuals: other mutations) and a total of 30 first-degree relatives without mutations were included in the analysis (Table 2). Here again, we did not identify patients or individuals with the REM behavior disorder that has been observed in prodromal neurodegenerative diseases. In the analysis, we focused on the 2 groups of *SOD1* gene carriers and *C9ORF72* gene carriers. The other mutation carriers were not analyzed because of the small sample size and heterogeneity.

**Figure 4:**
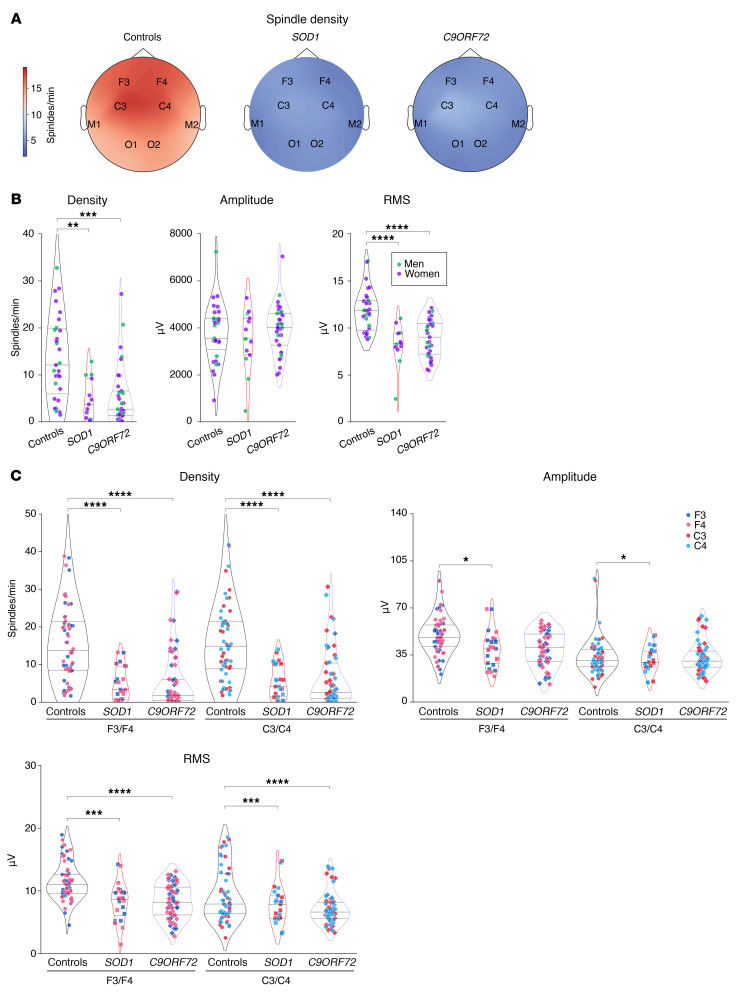
Sleep spindle alterations in presymptomatic ALS gene carriers. (**A**) Topographic map across all electrodes of sleep spindle density in controls and *SOD1* and *C9ORF72* presymptomatic gene carriers. (**B**) Quantification of sleep spindle density, amplitude, and RMS in controls and *SOD1* and *C9ORF72* presymptomatic gene carriers. **P_adj._ < 0.01, ***P_adj._ < 0.001, and ****P_adj._ < 0.0001, by 1-way ANOVA with FDR-BKY correction. (**C**) Quantification of sleep spindle density, amplitude, and RMS in controls and *SOD1* and *C9ORF72* presymptomatic gene carriers across F3/F4 and C3/C4 electrodes as indicated. *P_adj._ < 0.05, ***P_adj._ < 0.001, and ****P_adj._ < 0.0001, by Kruskal-Wallis test with Dunn’s multiple tests adjusted with FDR-BKY correction. Results with *P* > 0.05 are not indicated. Data are presented as medians and IQRs. Corrected *P* values are shown.

**Figure 5:**
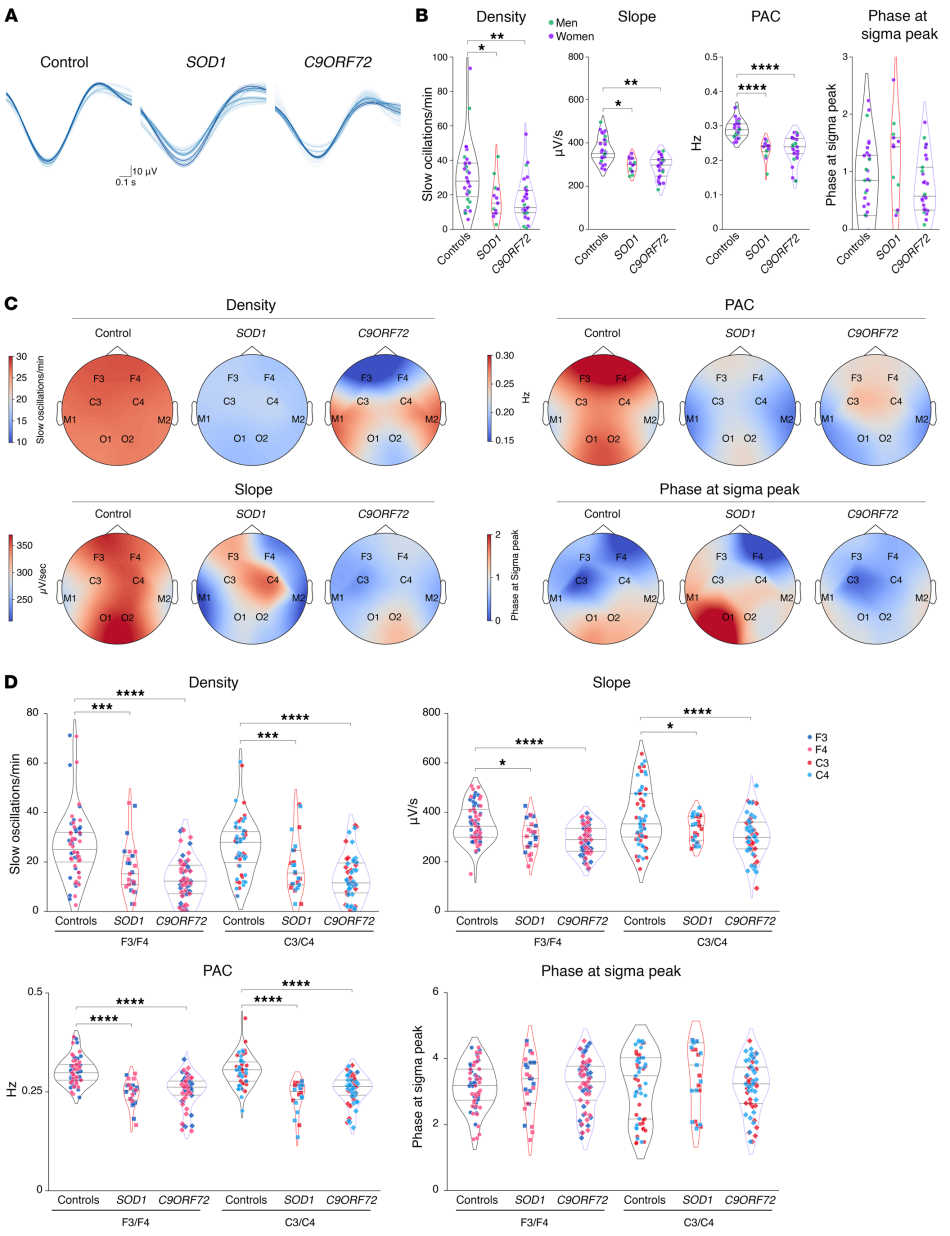
Slow oscillation alterations in presymptomatic ALS gene carriers. (**A**) Representative slow oscillations across all electrodes for a healthy individual, 1 presymptomatic *SOD1*, and 1 *C9ORF72* gene carrier. (**B**) Quantification of slow oscillation density, slope, PAC between sleep spindles and slow oscillations, and PSP in controls and *SOD1* and *C9ORF72* presymptomatic gene carriers. *P_adj._ < 0.05, **P_adj._ < 0.01, and ****P_adj._ < 0.0001, 1-way ANOVA with FDR-BKY correction. (**C**) Topographic maps across all electrodes of slow oscillation density, slope, PAC, and PSP in controls and *SOD1* and *C9ORF72* presymptomatic gene carriers. (**D**) Quantification of slow oscillation density, slope, PAC, and PSP across F3/F4 and C3/C4 electrodes in controls, *SOD1* and *C9ORF72* presymptomatic gene carriers as indicated. *P_adj._ < 0.05, ***P_adj._ < 0.001, and ****P_adj._ < 0.0001, by Kruskal-Wallis test with Dunn’s multiple tests adjusted with FDR-BKY correction. Results with *P* > 0.05 are not indicated. Data are presented as the median and IQRs. Corrected *P* values are shown.

Similarly to patients with ALS, presymptomatic *SOD1* and *C9ORF72* ALS gene carriers showed comparable reductions in sleep spindle density and RMS (Figure 4, A and B), with unchanged mean brain-wide amplitude (Figure 4B). The density and RMS of sleep spindles were decreased in both *SOD1* and *C9ORF72* gene carriers in both frontal and central electrodes (Figure 4C). Interestingly, *SOD1*, but not *C9ORF72*, gene carriers also displayed a mildly decreased amplitude of sleep spindle density in the frontal and central areas (Figure 4C). A brain-wide reduction in slow oscillations (Figure 5) and K-complexes (Supplemental Figure 3) was observed in presymptomatic gene carriers, similar to that observed in patients with ALS. As in patients with ALS, these results were not modified if individuals with intermediate AHI (5<AHI<20) and/or PMLSI (25<PMLSI<50) values were excluded from the analysis (Supplemental Figure 4). Thus, sleep microarchitecture was significantly affected in ALS gene carriers who did not yet show any signs of manifest disease.

Microarchitectural alterations of sleep patterns correlate with cognitive deficits. Previous research demonstrated a correlation between modifications in the microarchitecture of sleep and the presence of cognitive deficits (14, 16). Furthermore, there is a link between sleep spindles and motor memory consolidation (18). To determine whether this association could also be observed in our 2 cohorts, we correlated sleep microarchitecture parameters with cognitive function in patients with ALS and presymptomatic gene carriers, as well as with motor function in patients with ALS. We evaluated cognitive function using the Edinburgh Cognitive and Behavioral ALS Screen (ECAS) and motor function with the revised ALS functional rating scale (ALS-FRSr). In patients with ALS, cognitive testing was conducted 1–7 days after polysomnography in 58.5% of participants. the day before polysomnography, 13.2% of the participants underwent the ECAS test, and 26.4% were tested the following day. In the presymptomatic gene carrier cohort, the ECAS test was always administered in the morning after polysomnography. ALS-FRSr was recorded between 4 days before and 7 days after the sleep study.

**Figure 6:**
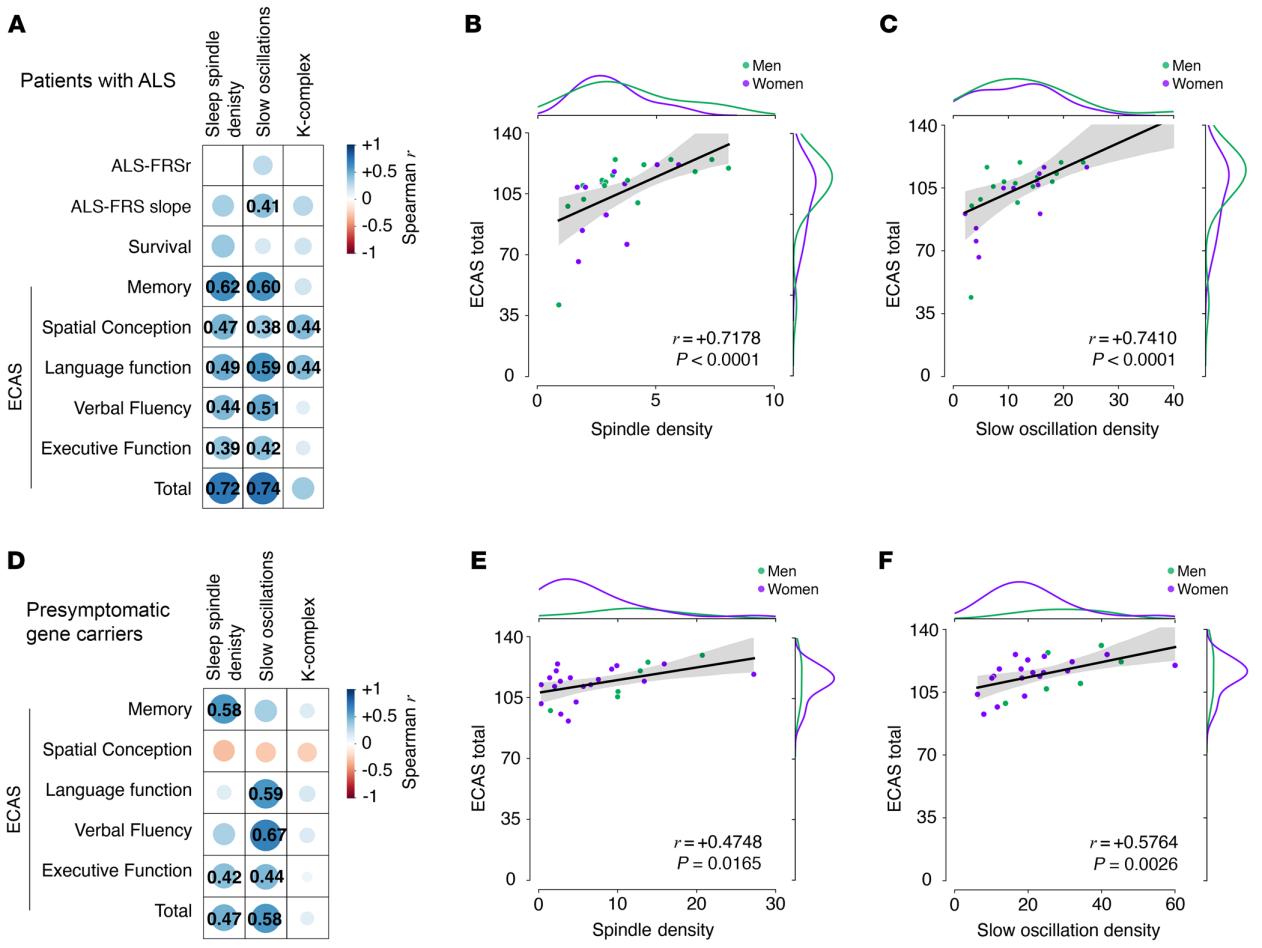
Correlation analysis between sleep microarchitecture, cognitive function, and motor function in patients with ALS and presymptomatic gene carriers. (**A**) Correlation matrix showing Spearman’s correlation coefficient *r* for each of the corresponding correlations performed for patients with ALS. Sleep spindle density, slow oscillation density, and K-complex density were correlated with ALS-FRSr, ALS-FRS slope, patient survival, and ECAS subscores as well as the total score for all patients with ALS. Only significant correlations are indicated with the numerical value of Spearman’s *r*. (**B** and **C**) Correlation between total ECAS scores and sleep spindle density (**B**) or slow oscillations density (**C**) for patients with ALS. (**D**) Correlation matrix showing Spearman’s correlation coefficient *r* for each of the corresponding correlations performed for presymptomatic gene carriers. Sleep spindle density, slow oscillation density, and K-complex density were correlated with ECAS subscores as well as the total score for all *SOD1* and *C9ORF72* gene carriers. Only significant correlations are indicated with the numerical value of Spearman’s *r*. (**E** and **F**) Correlation between total ECAS scores and sleep spindle density (**B**) or slow oscillation density (**C**) in presymptomatic gene carriers. Spearman’s *P* value was adjusted with FDR-BKY correction. Spearman’s correlation coefficient *r* and corrected *P* value are indicated. Side distribution represents sex distribution across both variables.

In patients with ALS, we observed a significant positive correlation after adjustment for multiple comparisons between total ECAS scores and either sleep spindle density or slow oscillation density (Figure 6, A–C). We also observed significant correlations between densities of sleep spindles or slow oscillation and various ECAS subscores (Figure 6A, Supplemental Figures 5 and 6, and Supplemental Table 1) including memory, language function, and verbal fluency subscores but not the ALS-FRSr score or its slope (Figure 6A and Supplemental Figures 5 and 6). In these patients, the correlations between cognitive assessment scores and K-complexes were noticeably weaker (Figure 6A and Supplemental Figure 7). In presymptomatic gene carriers, the densities of sleep spindles or slow oscillation correlated also with total ECAS scores (Figure 6, D–F, and Supplemental Table 2) as well as several ECAS subscores (Figure 6, D–F, and Supplemental Figures 8 and 9), while there were no correlations with K-complexes (Supplemental Figure 10). Thus, the present findings indicate that alterations in the sleep microarchitecture were associated with cognitive performance, particularly in relation to verbal fluency, language function and memory.

**Figure 7:**
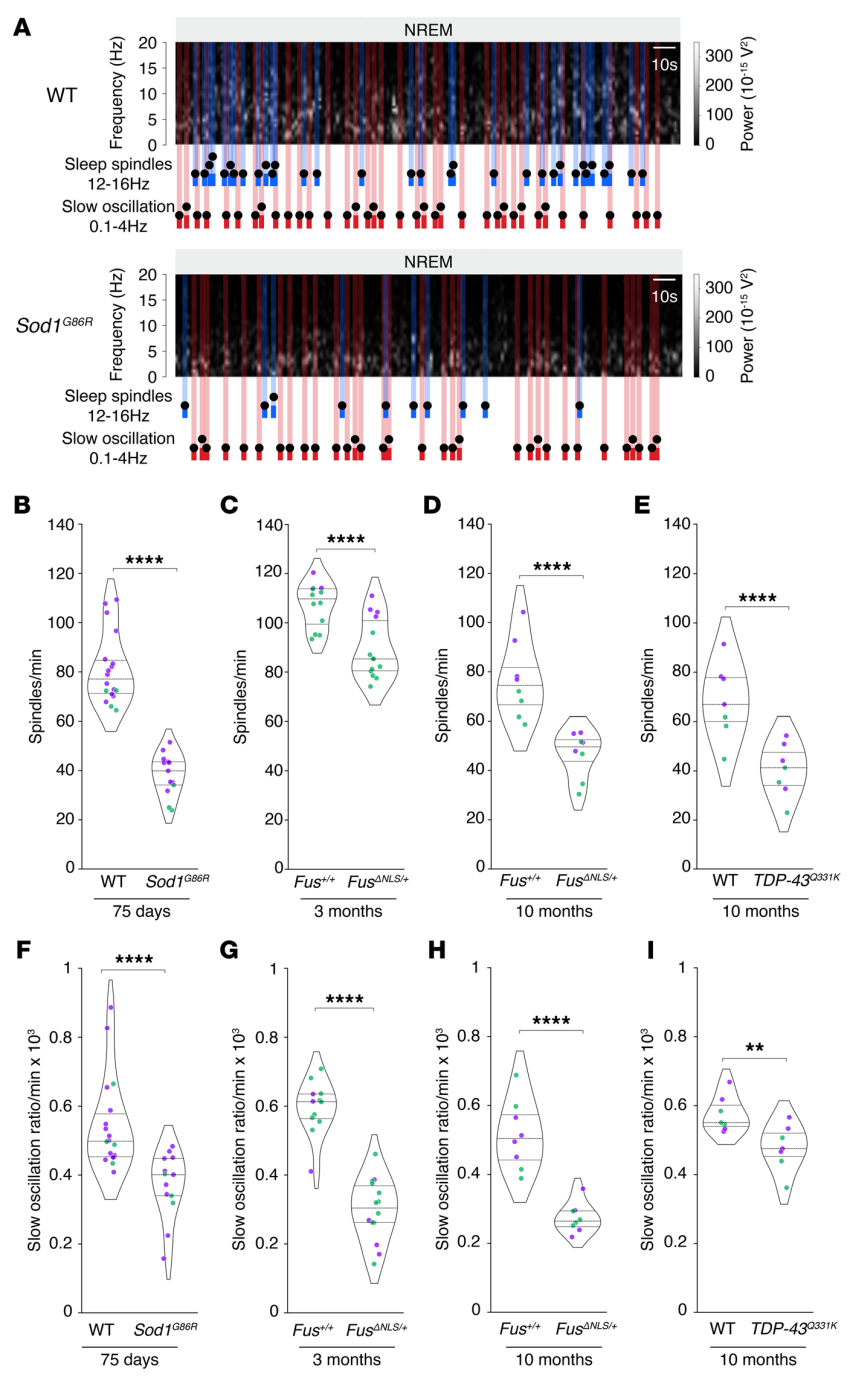
Sleep microarchitecture alterations in *Sod1^G86R^*, *Fus^ΔNLS/+^*, and *TDP-43^Q331K^* mice. (**A**) Representative spectrogram of *Sod1^G86R^* mice and their nontransgenic WT littermates at 75 days of age (prior to motor symptom onset). Sleep spindles are labeled in blue and slow oscillations in red on the spectrogram. (**B**–**I**) Quantification of sleep spindle density (**B**–**E**) and slow oscillation density (**F**–**I**) for *Sod1^G86R^* mice and their nontransgenic WT littermates at 75 days of age (**B** and **F**); for *Fus^ΔNLS/+^* mice and their WT littermates (*Fus^+/+^*) at 3 months of age (**C** and **G**, prior to motor symptom onset) or at 10 months of age (**D** and **H**); and for TDP-43^Q331K^ mice at 10 months of age (**E** and **I**). **P_adj._ < 0.01 and ****P_adj._ < 0.0001, by independent 2-tailed Student’s *t* test with Welch’s *t* test with FRD-BKY correction. Data are presented as medians and IQRs. Corrected *P* values are shown. Sleep spindle density: sex effect *Sod1*^G86R^ *P_adj._* = 0.0238, *Fus^ΔNLS/+^* 3 months *P_adj._* = 0.0183, *Fus^ΔNLS/+^* 10 months *P_adj._* = 0.0455, *TDP-43*^Q331K^ 10 months *P_adj._* = 0.0863. Slow oscillation density: Sex effect *Sod1*^G86R^ *P_adj._* =0.7128, *Fus^ΔNLS/+^* 3 months *P_adj._* = 0.2740, *Fus^ΔNLS/+^* 10 months *P_adj._* = 0.8112, *TDP-43*^Q331K^ 10 months *P_adj._* = 0.3648.

Three ALS models exhibit microarchitectural alterations of sleep patterns. Given the presence of sleep microarchitecture alterations in both patients with early-stage ALS and presymptomatic gene carriers, we sought to investigate whether these microarchitectural alterations are mirrored by findings in transgenic ALS mouse models. We studied 3 mouse models expressing distinct ALS-causing mutations, each with markedly disparate disease progression and different genetic backgrounds. The transgenic model *Sod1^G86R^* (on a FVB/N genetic background) is associated with severe and rapidly progressive motor symptoms (19, 20), whereas the *Fus^ΔNLS/+^* (21, 22) and TDP-43^Q331K^ models (on a C57BL/6 background) (23) are linked to a light-to-mild and late-onset phenotype (13). We used datasets previously acquired (13) from mouse cohorts implanted with intracortical electrodes, focusing on recordings taken during the presymptomatic phase. In parallel to our findings in patients with ALS and gene carriers, all 3 mouse models demonstrated substantial alterations in sleep spindles (Figure 7, A–E, and Supplemental Figure 11). It is noteworthy that there was a significant decrease in sleep spindle density in 3-month-old *Fus^ΔNLS/+^* mice (Figure 7C), despite showing no detectable alterations in sleep macroarchitecture at this stage (13). As in ALS patients and presymptomatic gene carriers, decreased sleep spindle density in all 3 mouse models was also accompanied by decreased densities in both slow oscillations (Figure 7, F–I) and K-complexes (Supplemental Figure 12). In all 3 models, we consistently observed microarchitectural alterations in both male and female mice. Thus, early changes in sleep microarchitecture were present in multiple ALS mouse models, mirroring the alterations observed in patients with early-stage ALS and presymptomatic gene carriers.

**Figure 8:**
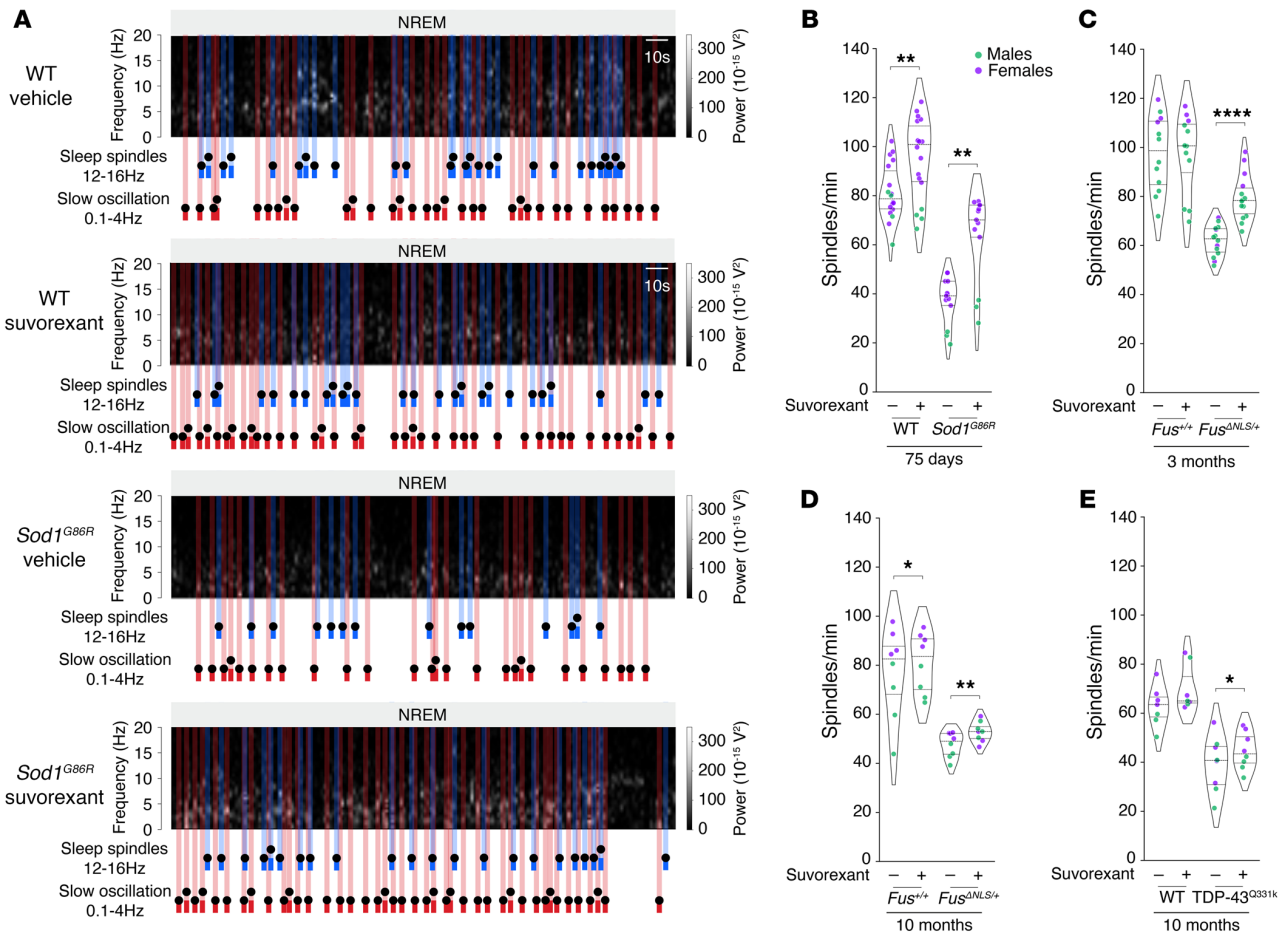
Improved sleep microarchitecture by suvorexant-treated *Sod1^G86R^*, *Fus^ΔNLS/+^*, and *TDP-43^Q331K^* mice. (**A**) Representative spectrogram of *Sod1^G86R^* mice and their nontransgenic WT littermates at 75 days of age (prior to motor symptom onset) administered either vehicle or suvorexant. Sleep spindles are labeled in blue and slow oscillations in red on the spectrogram. (**B**–**E**) Quantification of sleep spindle density in mice treated with either vehicle or suvorexant. The genotypes studied include *Sod1^G86R^* mice and their nontransgenic WT littermates at 75 days of age (**B**); in *Fus*^ΔNLS/+^ mice and their WT littermates (*Fus^+/+^*) at 3 months of age (**C**, prior to motor symptom onset) or at 10 months of age (**D**); and in TDP-43^Q331K^ mice at 10 months of age (**E**). NS *P_adj._* > 0.05, by 2-way ANOVA with Dunn’s test and FDR-BKY correction. Genotype effect (not indicated on figure) *Sod1*^G86R^ *P_adj._* < 0.0001 *Fus^ΔNLS/+^* 3 months *P_adj._* < 0.0001, *Fus^ΔNLS/+^* 10 months *P_adj._* < 0.0001, *TDP-43*^Q331K^ 10 months *P_adj._* < 0.0001; sex effect *Sod1*^G86R^ *P_adj._* < 0.0001, *Fus^ΔNLS/+^* 3 months *P_adj._* = 0.4584, *Fus^ΔNLS/+^* 10 months *P_adj._* = 0.3032, *TDP-43*^Q331K^ 10 months *P_adj._* = 0.1522. Data are presented as medians and IQRs. Corrected *P* values are shown. *P_adj._ < 0.05, **P_adj._ < 0.01, and ****P_adj._ < 0.0001.

Sleep spindle defects are rescued by an orexin antagonist. We previously showed that sleep alterations in ALS mouse models can be fully rescued through administration of suvorexant, a dual orexin receptor antagonist. We reanalyzed the previous datasets from studies in which suvorexant or its vehicle was administered orally to mice at the onset of the inactive period (i.e., during the day in mice). Single administration of suvorexant increased sleep spindle density in all 3 mouse models to near-WT levels, with a more pronounced effect in females, whereas efficacy was diminished in aged 10-month-old mice (Figure 8 and Supplemental Figure 13). We observed similar restorative effects for slow oscillations and K-complexes (Supplemental Figure 14). MCH supplementation had similar, yet blunted, effects on all sleep microarchitecture events in both *Sod1^G86R^* mice (Supplemental Figure 15) and *Fus^ΔNLS/+^* mice (Supplemental Figure 16). In all, our results suggest that increased orexinergic tone is causally related to sleep microarchitectural defects in both ALS mouse models and patients.

## Discussion

In this study, we demonstrate that the disruption of sleep microarchitecture in ALS was profound and brain wide and preceded the onset of motor symptoms in both humans and mouse models.

Alterations in sleep microarchitecture in ALS affect multiple sleep-related EEG features, including sleep spindles, slow oscillations, and K-complexes. In ALS patients without respiratory impairment, as well as in presymptomatic ALS gene carriers, we observed reduced densities for all 3 EEG signals compared with their respective controls. The decrease in density was brain wide, and we did not observe disruption patterns confined to isolated central or frontal regions. We also observed similar alterations in sleep microarchitecture in 3 mouse models of ALS. These defects in microarchitecture were more pronounced and appeared earlier in mouse models than macroarchitectural abnormalities (13). Indeed, while we previously did not observe increased wakefulness or decreased NREM in 3-month-old *Fus^ΔNLS/+^* mice, there was a loss of sleep spindles, slow oscillations, and K-complexes at this age. These findings need to be confirmed in patients with *FUS* mutations but could be used as some of the earliest detectable changes in ALS pathophysiology, as they precede other symptomatic and sleep macroarchitecture alterations observed in that particular mutation.

Disruption of sleep EEG features, as we describe here in ALS, is widely observed in neurological and neurodegenerative diseases. Loss of sleep spindles, particularly spindle density, and loss of slow oscillations have been consistently observed in Alzheimer’s disease (24–26) and Parkinson’s disease (27–29) and in other neurological diseases such as temporal lobe epilepsy (30) and schizophrenia (31–33). Remarkably, this was not observed in patients with attention-deficit/hyperactivity disorder (ADHD) or post-traumatic stress disorder (PTSD), or in most patients with autism spectrum disorder (31). Sleep spindle density also decreases with age (34), suggesting that accelerated brain aging could account for some of our results in patients with ALS, both in terms of microarchitectural defects (the current study) or macroarchitectural changes (13). Furthermore, as decreased sleep spindle density is observed in several neurological and neurodegenerative conditions, the current observation is unlikely to be used as diagnostic. Nevertheless, sleep spindles and slow oscillations are quantifiable parameters, and their disruption appears early, in the prodromal stage of the disease. Therefore, it is reasonable to hypothesize that sleep EEGs may contain hallmarks that could be explored for prognostic purposes.

There are 2 limitations of our current description of sleep microarchitectural defects. First, we present evidence of presymptomatic defects only in fALS, in both presymptomatic gene carriers and mouse models. We do not have access to prediagnostic polysomnography in sporadic ALS (sALS) and can only assume that our observation in fALS extends to sALS. Second, our current study is cross-sectional and lacks longitudinal follow-up. Consequently, we are currently unable to determine whether these phenotypes worsen as symptom onset approaches or as the disease progresses. Future studies should include longitudinal polysomnography in presymptomatic gene carriers to determine the dynamics of sleep macro- and microarchitecture and their possible role as prognostic markers of phenoconversion.

What might be the consequences of defects in sleep microarchitecture? It has been documented that sleep spindles and slow oscillations are causally involved in memory consolidation and executive functions (14, 16). In patients with Parkinson’s or Alzheimer’s disease, the severity of sleep spindle disruption has been linked to memory and cognitive impairments (24–29). Complementing these findings, we observed strong correlations between cognitive scores and sleep spindles and slow oscillations in our 2 cohorts. These correlations were stronger than previously reported associations between sleep stages and cognitive function in the same cohorts (13). Whether loss of sleep spindles affects motor progression in ALS remains unknown. Notably, sleep spindles are highly associated with motor adaptation (35) and motor memory consolidation (18, 36–39), and disruption of this functional balance could potentially accelerate or exacerbate motor decline. Longitudinal studies in patients and gene carriers, as well as experimental rodent studies, may help address this question.

Defects in sleep microarchitecture may arise from multiple causes, which our study does not definitively distinguish. First, defects in intracortical circuits could impair slow oscillations, sleep spindles, and K-complexes, as these EEG patterns originate and propagate locally within cortical networks, particularly the frontal part (40–45). In this context, impaired frontal lobe function in ALS (46, 47) may contribute to the abnormalities observed. Second, these defects could result from dysfunction in subcortical structures such as the thalamus. Sleep spindles originate in the thalamus (14, 16), which also plays a key role in modulating the timing, coherence, and spindle nesting of slow oscillations (48, 49). Thalamic atrophy has been documented in ALS, particularly in patients carrying the *C9ORF72* gene (50–55), and alterations of the thalamo-cortical pathway have been observed in both patients with sALS (56, 57) and presymptomatic *C9ORF72* gene carriers (58). These thalamic alterations have been linked to faster progression in sALS (59) and a higher risk of phenoconversion in *C9ORF72* ALS (60). Thalamic involvement is considered an early biomarker of ALS, contributing to both motor and cognitive deficits in sporadic cases (61–64), and is described as an early event, i.e., stage 2 in the Braak staging system of TDP-43 pathology spread (65, 66). Third, impairments in monoaminergic pathways, particularly acetylcholine (67–70) or norepinephrine (71–73) pathways, could be the origin of sleep microarchitecture alterations. This would align with previous observations of acetylcholine defects (70) or norepinephrine dysfunction (74) in ALS. Finally, part of the observed microarchitectural changes may be caused by hypothalamic defects, such as orexin or MCH signaling alterations. Our results show that orexin antagonism or MCH supplementation can rescue the loss of sleep spindles and slow oscillations in several ALS mouse models, strongly implicating the hypothalamus. This aligns with reported MCH neuron loss in ALS postmortem tissues (75) and several studies indicating orexin pathway alterations in patients with ALS and mouse models (13, 76, 77). These mechanisms are not mutually exclusive, as thalamic reticular nucleus neurons are sensitive to orexin (78, 79) and are also highly modulated by cholinergic or noradrenergic innervation during sleep (72, 80–82). Our findings do not establish a direct causal relationship between MCH/orexin signaling and microarchitecture sleep defects. It remains possible that sleep dysfunctions originate in other brain regions such as the cortex, locus coeruleus, or thalamus and are corrected by MCH or suvorexant. Elucidating the mechanisms behind sleep microarchitecture defects will require further experimental investigation.

In summary, our study identifies alterations in sleep EEGs as an early biomarker of ALS that is detectable many years before symptom onset in presymptomatic gene carriers and correlates with cognitive decline. Uncovering the underlying mechanisms may offer critical insights into the earliest pathophysiological events in ALS. Further longitudinal studies are needed to evaluate whether sleep microarchitectural alterations can serve as prognostic markers for phenoconversion and disease progression, especially given the easy testing set-up in clinical practice. Preclinical evaluation of chronic administration of dual orexin receptor antagonists is now required to determine their safety in ALS models and their potential translational relevance for improving sleep, mitigating cognitive deficits, preventing weight loss, and slowing motor symptom progression, and will likely justify future human clinical trials.

## Methods

### Sex as a biological variable.

Our study involved both male and female individuals, for both the human studies and experimental mouse models. Sex is indicated in each figure and was considered a biological variable for statistical analysis. The effect of sex is reported in the figure legends.

### Patients and participants.

Patients with ALS were recruited from the inpatient and outpatient clinics of the Neurology Department of the University Hospital of Ulm (Ulm, Germany). The inclusion criteria for patients with ALS included a definitive diagnosis of ALS based on the revised El Escorial criteria (83). Presymptomatic carriers of fALS genes were recruited through the study center of the University Neurology Clinic, which provides longitudinal follow-up and counseling to first-degree relatives of patients with confirmed fALS. Controls were recruited from the general population of the Neurology Clinic and were matched to patients with ALS on the basis of age, sex, and geographical location; the requirement for inclusion in this group was the absence of neurodegenerative diseases. All individuals in the control group had no history of ALS or fALS.

The participants’ medical history was documented. For patients with ALS, the ALS-FRSr and characteristics of disease progression were documented (site of first paresis/atrophy, date of onset). All participants also completed validated daytime sleepiness and sleep quality questionnaires, namely the Epworth sleepiness scale (ESS) (84) and the Pittsburgh sleep quality index (PSQI) (85). Current medication for all participants were documented, and no exclusion criteria related to medication use were applied that would have prevented participation.

### Patients’ inclusion process.

The same exclusion criteria used by Guillot et al. (13) were applied and are summarized in Figure 1. Participation in the study was possible in this cohort for both patients with sALS and those with fALS. Exclusion criteria were designed to exclude all possible circumstances that might otherwise alter sleep architecture. For this reason, participants who had an AHI higher than 20 per hour, or participants who had a periodic limb movement index (PLMSI) above 50 per hour were excluded. In particular, we intended to exclude respiratory insufficiency in patients with ALS. Respiratory insufficiency develops earlier or later in the progression of ALS, depending on the individual course, but is generally present in advanced stages and is known to influence sleep architecture (86). For this reason, patients with ALS underwent transcutaneous capnometry in addition to polysomnography. Capnometry results had to be normal, or nocturnal hypercapnia would result in exclusion from the study.

### Inclusion process of first-degree relatives of patients with fALS.

The same exclusion criteria applied by Guillot et al. (13) were followed. We enrolled first-degree relatives of patients with fALS for whom the ALS-causing mutation was known. The participants were examined according to the study protocol, and genotyping was also performed. Neither the participants nor the study personnel were informed of the genetic results at the time of the examinations. After the study visits, participants were assigned to the group of presymptomatic gene carriers or the control group with negative genetic findings based on their genetic results. The participants could only learn the result of the genetic test if they had completed the legally required genetic counseling appointments (in accordance with the German Genetic Diagnostics Act).

### Neuropsychological assessment.

Trained neuropsychologists measured cognition with the German version of the ECAS (87–89). The ECAS addresses the cognitive domains of language function, verbal fluency, executive functions (ALS-specific functions), and memory and visuospatial functions (ALS-nonspecific functions). Age- and education-adjusted cut-offs were used (89). Behavioral changes were assessed by patient caregiver/first-degree relative interviews on disinhibition, apathy, loss of sympathy/empathy, perseverative/stereotyped behavior, hyperorality/altered eating behavior, and psychotic symptoms. Since the participants in the ALS patient cohort were hospitalized, the neuropsychological examination was conducted in the days before or after the polysomnography, with a maximum interval of 1 week. The participants of the presymptomatic gene carrier cohort underwent the neuropsychological examination the morning after the polysomnography.

### Electroencephalography in patients and controls.

All participants — patients with ALS, healthy individuals, presymptomatic fALS gene carriers, and fALS control individuals — underwent a 1-night full polysomnography, which was performed at the inpatient sleep laboratory of the Department of Neurology at Ulm University Hospital and involved monitoring of various physiological parameters including EEG, surface electromyogram (EMG), electrooculogram (EOG), respiratory effort and flow, pulse, and oxygen saturation. To minimize bias, conditions were standardized within each cohort: presymptomatic gene carriers followed a protocol with arrival in the morning, polysomnography at night, and neuropsychology the next day, and ALS patients along with their controls underwent polysomnography during a single night of a multi-day inpatient stay. All measurements were conducted according to the criteria of the American Academy of Sleep Medicine (AASM) guidelines (90, 91). The EEG electrodes were placed according to the international 10–20 system. The following electrodes were used for each participant: Fz, C3, C4, Cz, P3, P4, Pz, O1, O2, M1, and M2. The sampling rate was 512 Hz in each case. The EEG montage was in accordance with the general recommendation of the AASM guidelines, with contralateral referencing to the mastoid electrode, i.e., F3–M2, C3–M2, O1–M2, and F4–M1, C4–M1, O2–M1. A “lights-off” marker was placed in each recording to indicate the individual point in time when the participant turned off the lights and attempted to sleep. In order to accommodate sleep times as closely as possible to the chronotype, participants were allowed to freely choose the start time of the examination, but the latest time was midnight. The occurrence of a REM sleep behavior disorder was evaluated in all individuals using previously defined criteria (17).

### Sleep analyses in patients and controls.

Analyses were performed using available Python packages (only compatible with Python 3.10 or newer, Python Software Foundation. Python Language Reference, version 3.12. http://www.python.org), relying on the MNE package (92). EEG preprocessing was performed following the guidelines of Guillot SJ et al. (13).

Briefly, recordings were first deidentified using the open-source Prerau Lab EDF Deidentification Tool (version 1.0; 2023) in Python (Prerau Lab EDF Deidentification Tool retrieved from https://sleepeeg.org/edf-de-identification-tool) and were then notch filtered to remove the 50 Hz powerline. Independent component analysis was performed to remove all remaining artifacts from the signal (93–97).

Analyses of sleep spindles, slow oscillations, and K-complexes were performed on all electrodes (41, 98, 99), and outliers were removed using an isolation forest algorithm (100). K-complex analysis was limited to the sensorimotor cortex (C3), which is known to be impaired in ALS, using MNE and SciPy packages (100, 101). For the sleep spindles, their density (number of sleep spindles per minute of NREM2/3 sleep), amplitude (peak-to-peak amplitude of the detrended sleep spindle), and RMS were measured, with the frequency bands set to 12–15 Hz and required to last 0.5–2 seconds. For slow oscillations, their density (number of slow oscillations per minute of NREM2/3), slope (slope between the negative peak and the midpoint of the slow oscillation), phase-amplitude coupling (PAC) (slow oscillations sleep spindle–normalized PAC within a 2-second epoch centered around the negative peak of the slow oscillation), and phase at sigma peak (phase of the slow oscillation when the sigma peak is reached within a 2-second epoch centered around the negative peak of the slow oscillation) were analyzed, with the frequency bands set to 0.3–1.5 Hz and amplitude below 150 μV. For the K-complex assessment, we analyzed the density (number of K-complexes per minute of NREM2), with the frequency bands set to 0.3–1 Hz and amplitude between 100 and 350 μV. Topographic maps were generated using the MNE and YASA packages (101). All analyses were performed following the AASM’s guidelines (102).

### Electrocorticography analysis in mice.

Data were extracted from the NeuroScore software for sleep and seizure analysis 3.4 (Data Science International) and used in combination with already available Python packages (Python Software Foundation. Python Language Reference, version 3.12. http://www.python.org) to further process the data.

Sleep spindles, slow oscillations, and K-complexes were automatically detected using publicly available pipelines (103, 104). The signal was first band-pass filtered at 1–45 Hz, and the sigma power (12–16 Hz) was calculated on a 200 ms Hamming window followed by a short-term Fourier transform (STFT) with the same window length. The occurrence of sleep spindles was identified when the smoothed absolute sigma power within the 12–16 Hz range exceeded 0.2 of the total power observed in the broadband frequency range of 0.1–45 Hz. This signifies that at least 20% of the total signal power must be within the specified sigma band. 

 Equation 1


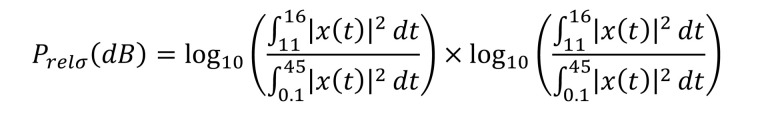


aFor slow oscillations, the signal was first band-pass filtered at 0.1–45 Hz, and the low delta power (0.1–2 Hz) was calculated on a 400 ms Hamming window. AUCs were calculated using Simpson’s rule derived from the delta band (A*_so_*) and the total power broadband frequency range (B*_PSD_*). The ratio of these 2 AUCs was then obtained, providing the slow oscillations ratio.

 Equation 2


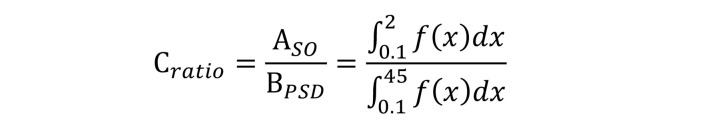


For K-complexes, the signal was first band-pass filtered at 0.1–45 Hz, and the sigma power was calculated on a 400 ms Hamming window. The signal was first band-pass filtered at 1–45 Hz, and the low delta power (0.3–1 Hz) was calculated on a 200 ms Hamming window followed by an STFT with the same window length. The occurrence of K-complexes was identified when the STFT within the 0.3–1 Hz range exceeded the mean STFT of the same frequency range.

 Equation 3


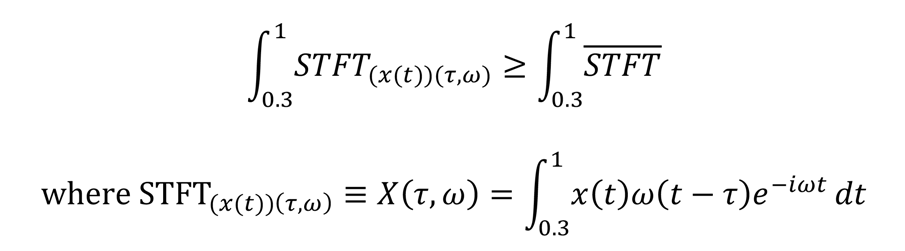


### Statistics.

G*Power software (version 3.1.9.6 for macOS; 2023) was used to determine the sufficient sample size needed to achieve significant statistical power using an a priori 2-tailed Student’s *t* test coupled with a linear bivariate regression (105, 106).

Prior to any statistical analysis, normality and homoscedasticity were both tested respectively with a Shapiro-Wilk test (107) and Bartlett’s test (108).

For comparisons between 2 groups, an independent 2-tailed Student’s *t* test was performed using Pingouin (109), with Welch’s *t* test correction, from SciPy, as recommended by Zimmerman (110). A large Cauchy scale factor was incorporated, given the considered effect size (111). When data violated normality or heteroscedastic a Mann-Whitney *U* test was performed using SciPy (100).

Follow-up analyses were performed using a paired *t* test from SciPy (100) or a Wilcoxon-Mann-Whitney rank-sum test from statsmodels (112) when normality was not met. *P* values were then adjusted using FDR–Benjamini, Krieger, and Yekutieli (FDR-BKY) correction.

For comparisons among 3 or 4 groups, a 1- or 2-way ANOVA was performed using the Pingouin (109) toolbox. For both ANOVA models, a multiple-comparison test with FDR-BKY correction was applied. If normality or heteroscedastic assumptions were not met, a Kruskal-Wallis test from SciPy (100) followed by Dunn’s multiple-comparison test with FDR-BKY correction was performed using scikit–post hocs (113) instead of a 1-way ANOVA. For the 2-way ANOVA, a generalized least-squares model was fitted using statsmodels (112), followed by Dunn’s multiple-comparison test and FDR-BKY correction using scikit–post hocs (113). We evaluated whether a sex-specific effect was present in all our analyses by performing a 2-way ANOVA followed by a multiple-comparison test with FDR-BKY correction for both sexes. Sex was self-reported in both ALS cohorts.

Spearman’s correlation coefficient from SciPy (100), was used for correlations in nonparametric data.

Data are presented as violin plots with all points and expressed as the median ± IQR. Visualizations were generated using Seaborn and Matplotlib packages (114). Results were deemed significant when the adjusted (adj.) *P* value was less than 0.05. Here, only corrected *P* values (adj. *P* values) are shown.

### Study approval.

The study involving the ALS patient cohort was approved by the ethics committee of the University of Ulm (reference 391/18). The study in presymptomatic carriers received approval by the same ethics committee of the University of Ulm (reference 68/19), in accordance with the ethics standards of the current version of the revised Helsinki Declaration. All participants provided written informed consent prior to enrollment.

Mouse experiments were performed in full compliance with Directive 2010/63/EU and new Regulation (EU) 2019/1010, and the project was reviewed and approved by the Ethics Committee of the University of Strasbourg and the French Ministry of Higher Education, Research and Innovation (decree no. 2013-118, February 1, 2013). All procedures including animal care and surgery and the datasets used in this study have been previously described (13).

### Data availability.

Additional data are available upon request to the corresponding authors. All numerical data are provided in the Supporting Data Values file.

## Author contributions

CL, SJG, ACL, MB and LD conceptualized the study. CL, SJG, DL, LTB, AK, PW, JD, KK, HPM, JK, LW, MB performed the experiments. CL, SJG, ACL, MB, LD analyzed the results. CL, SJG, LD generated visualization of the results. SJG, SDC, ACL, MB, LD provided resources. ACL, FR, LD acquired funding. AK, PW, JD, FR, ACL, MB, LD were involved in project administration. ACL, MB, LD supervised the project. CL, SJG, MB, LD wrote the original draft of the manuscript, and all authors reviewed, edited, and approved the manuscript.

## Funding support

- Fondation Anne-Marie et Roger Dreyfus (hosted by Fondation de France) (to SJG).
- Charcot Stiftung (to CL).
- Agence Nationale de la Recherche (ANR-24-CE37-4064).
- Interdisciplinary Thematic Institute NeuroStra (as part of the ITI 2021-2028, Idex Unistra ANR-10-IDEX-0002, ANR-20-SFRI-0012).
- Fondation Thierry Latran.
- Association Francaise de Recherche sur la Sclérose Latérale Amyotrophique, Association Française contre les Myopathies (AFM-Téléthon, no. 28944).
- TargetALS and JPND (HiCALS project).

## Supplementary Material

Supplemental data

ICMJE disclosure forms

Supporting data values

## Figures and Tables

**Table 1:**
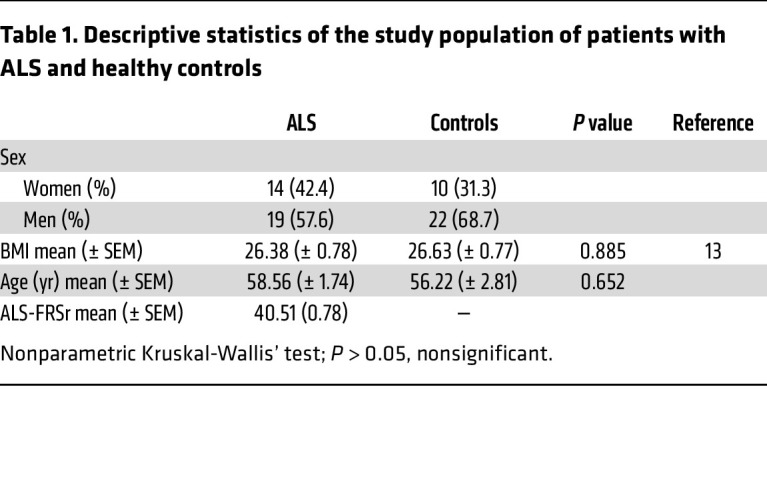
Descriptive statistics of the study population of patients with ALS and healthy controls

**Table 2:**
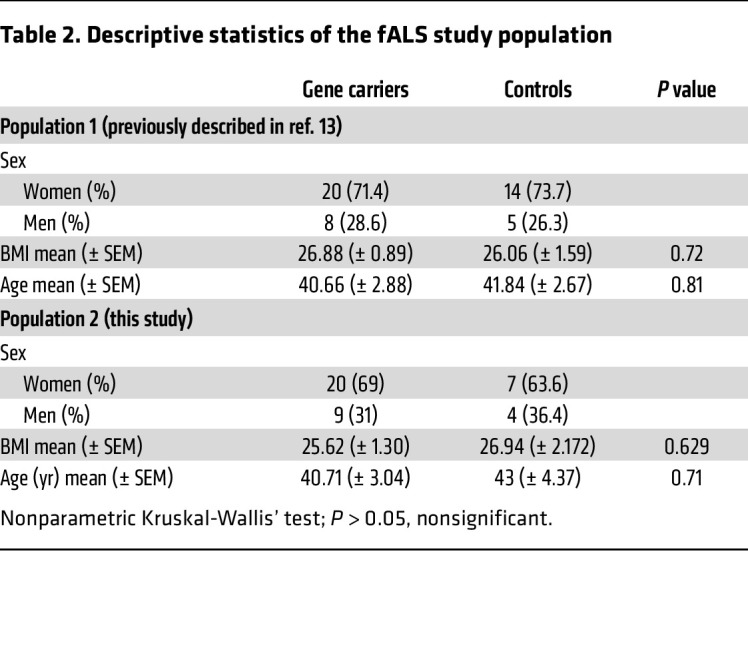
Descriptive statistics of the fALS study population

## Version 1 11/06/2025

In-Press Preview

## Version 2 01/02/2026

Electronic publication

## Version 3 01/12/2026

Loaded missing equation files
