# Supplementary material for: Early brain-wide disruption of sleep microarchitecture in amyotrophic lateral sclerosis
Source: J Clin Invest. 2025 Nov 6;136(1):e194555. doi: 10.1172/JCI194555 (PMC12721911; doi:10.1172/JCI194555)
Supplement: Supplemental data [file jci-136-194555-s014.pdf]

Supplementary figures and legends

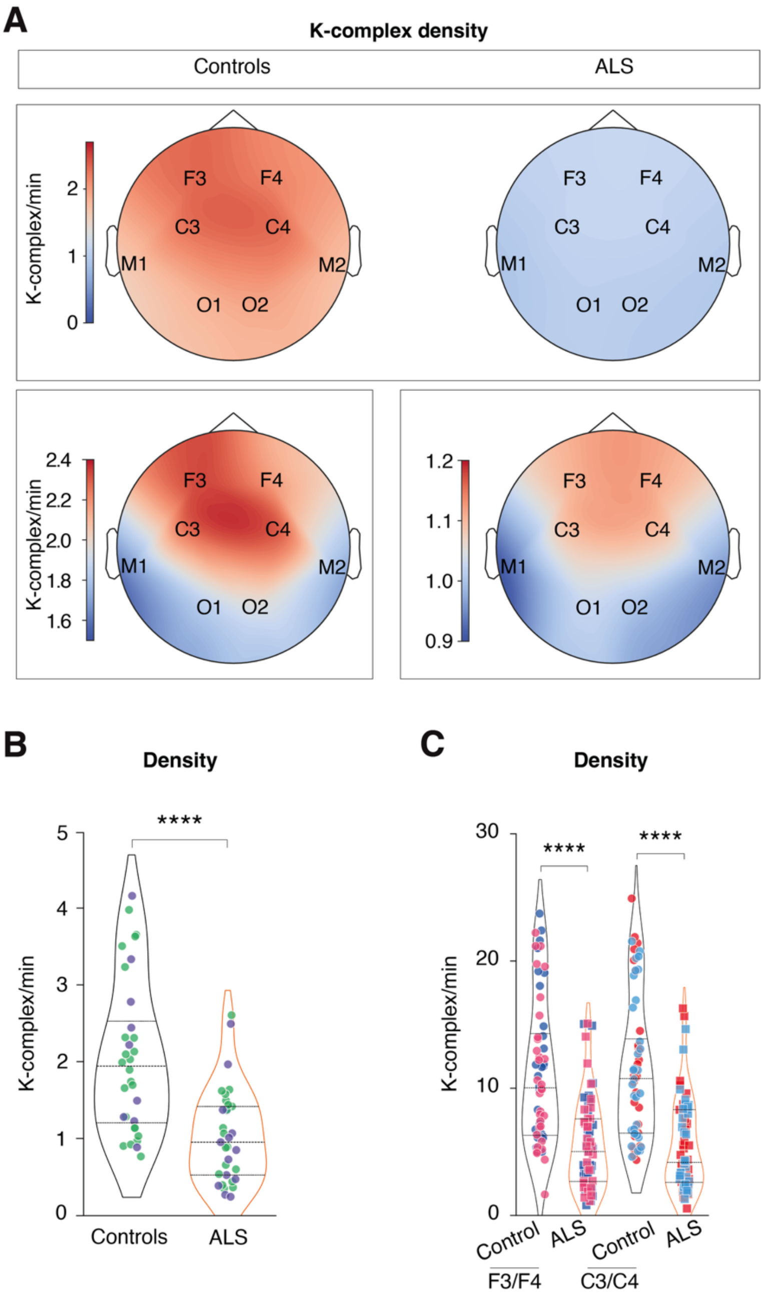

Supplementary Figure 1: K-complex alterations in early ALS patients.

(**A**) Topographic map across all electrodes of K-complex density in controls and ALS patients. To illustrate topographical differences, the maps were individually rescaled in the bottom panels as indicated.

(**B**) Quantification of K-complex density in controls and ALS patients. **** adj. $p_{value}<0.0001$, independent Student's t-test with Welch's t-test correction. Men are shown in green and women in purple.

(**C**) Quantification of K-complex density across F3/F4 and C3/C4 electrodes as indicated. **** adj. $p_{value}<0.0001$, Kruskal-Wallis test with Dunn's multiple tests adjusted with FDR-BKY correction.

Data are presented as medians and interquartile ranges. Corrected p_{value} are shown.

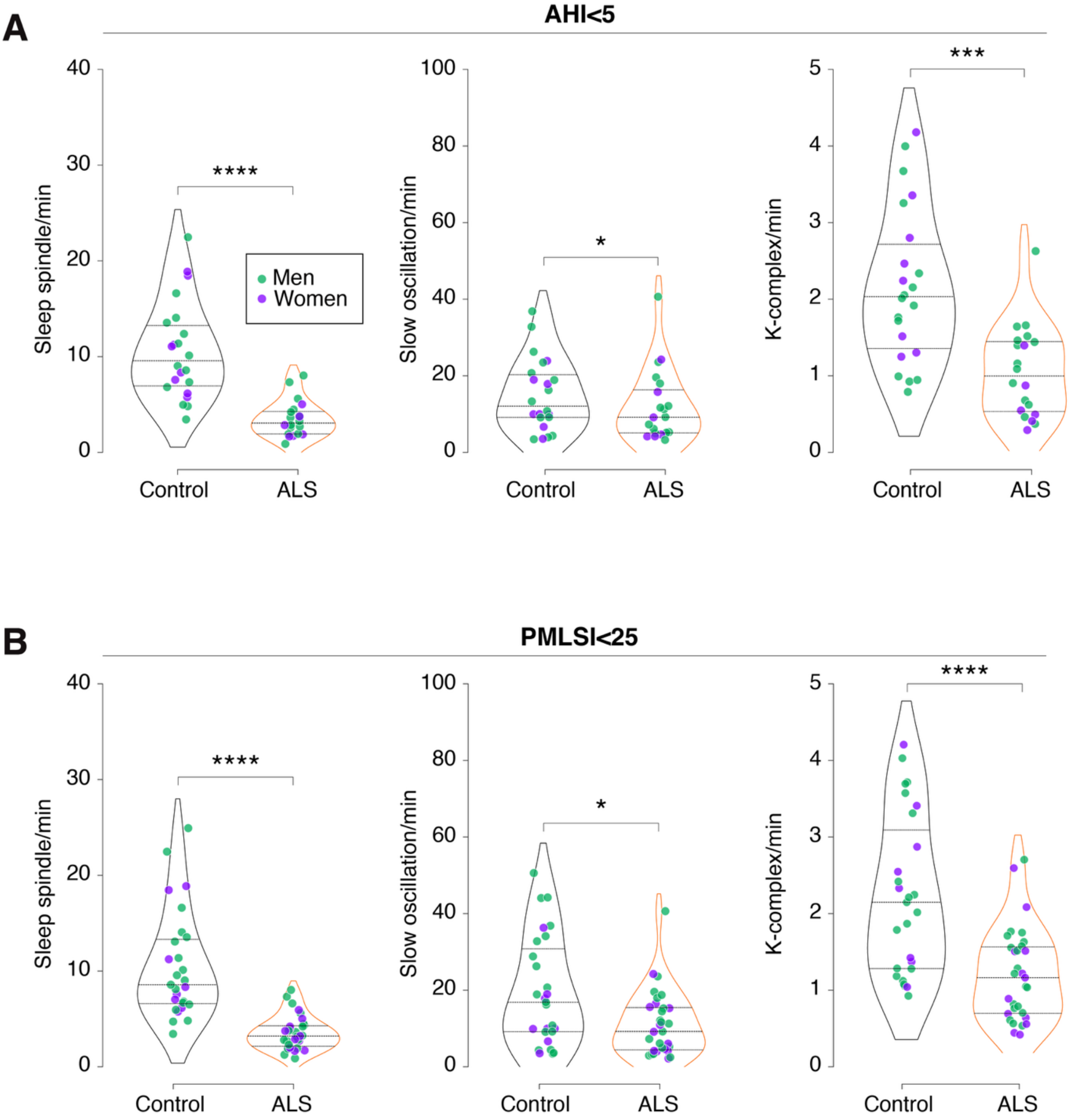

Supplementary Figure 2: Sleep microarchitecture in patients with AHI<5 and PMLSI<25
Quantification of sleep spindle density, slow oscillation density and K complex density in patients and controls with AHI<5 (A) or PMLSI<25 (B). * adj. p_{value}<0.05, *** adj. p_{value}<0.001 **** adj. p_{value}<0.0001, independent Student's t-test with Welch's t-test correction. Men are shown in green and women in purple.

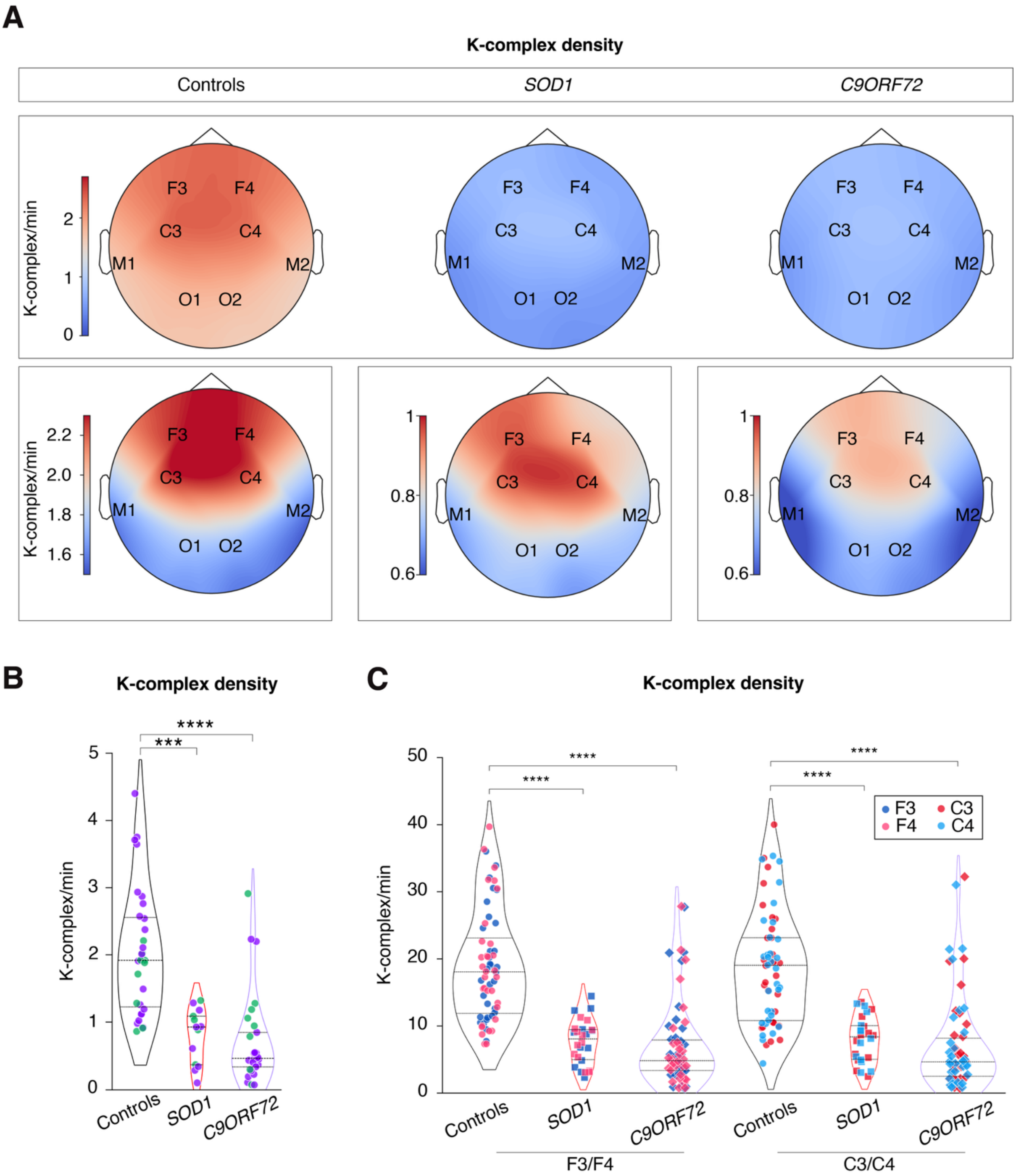

Supplementary Figure 3: K-complex alterations in presymptomatic ALS gene carriers. (**A**) Topographic map across all electrodes of K-complex density in controls, *SOD1* and *C9ORF72* presymptomatic gene carriers. To illustrate topographical differences, the maps were rescaled and shown using individual scales as indicated on the bottom panels.

(**B**) Quantification of K-complex density in controls, *SOD1* and *C9ORF72* presymptomatic gene carriers. *** adj. $p_{value}<0.001$, **** adj. $p_{value}<0.0001$, One-way ANOVA with FDR-BKY correction. Men are shown in green and women in purple.

(**C**) Quantification of K-complex density across F3/F4 and C3/C4 electrodes in controls, *SOD1* and *C9ORF72* presymptomatic gene carriers as indicated. **** adj. $p_{value}<0.0001$, Kruskal-Wallis test with Dunn's multiple tests adjusted with FDR-BKY correction.

Results with $p_{value}>0.05$ are not indicated. Data are presented as medians and interquartile ranges. Corrected p_{value} are shown.

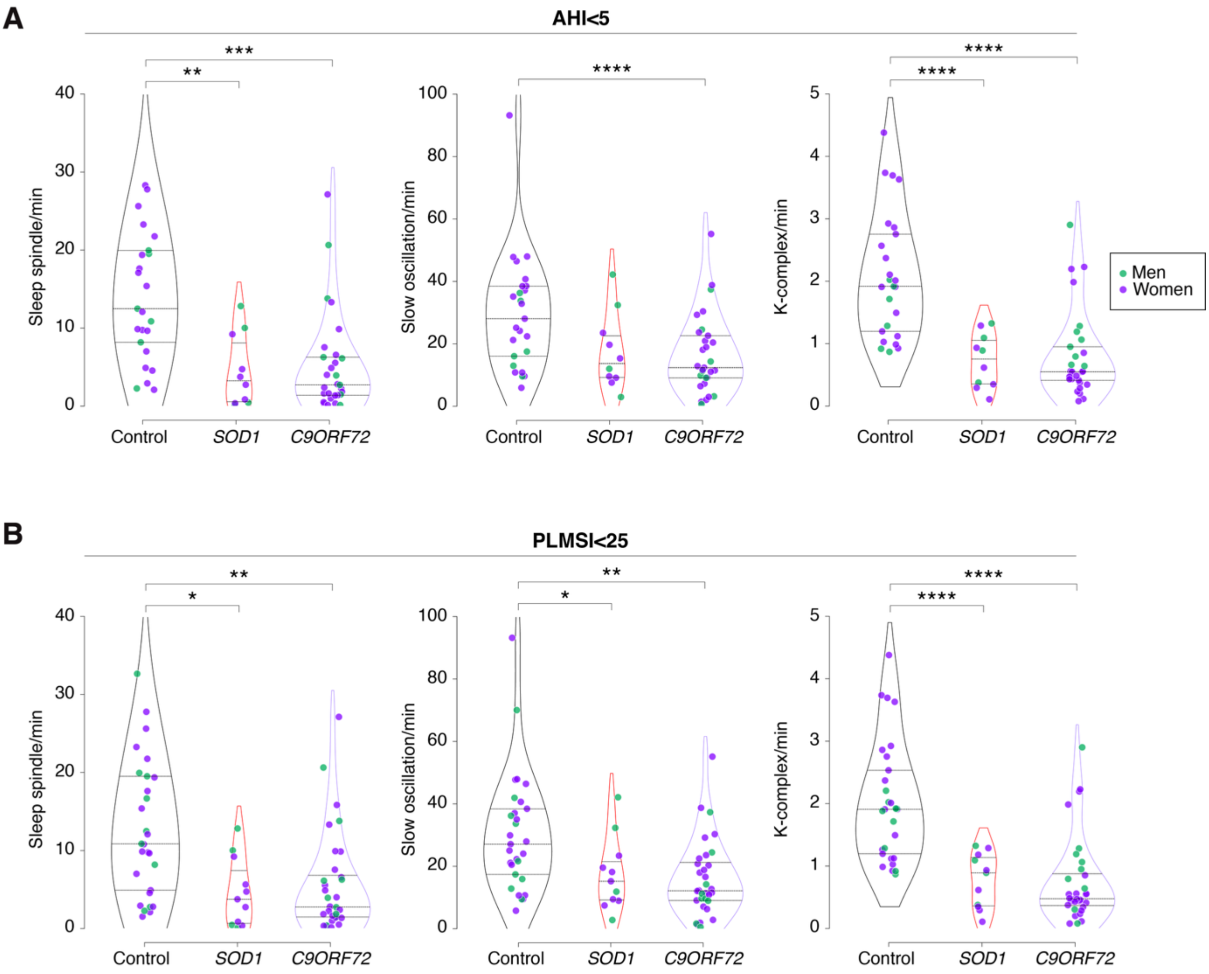

Supplementary Figure 4: Sleep microarchitecture in presymptomatic ALS gene carriers with AHI<5 and PMLSI<25

Quantification of sleep spindle density, slow oscillation density and K complex density in in controls, *SOD1* and *C9ORF72* presymptomatic gene carriers with AHI<5 (A) or PMLSI<25 (B). * adj. $p_{value}<0.05$, ** adj. $p_{value}<0.001$ **** adj. $p_{value}<0.001$ **** adj. $p_{value}<0.0001$, Kruskal-Wallis test with Dunn's multiple tests adjusted with FDR-BKY correction. Men are shown in green and women in purple.

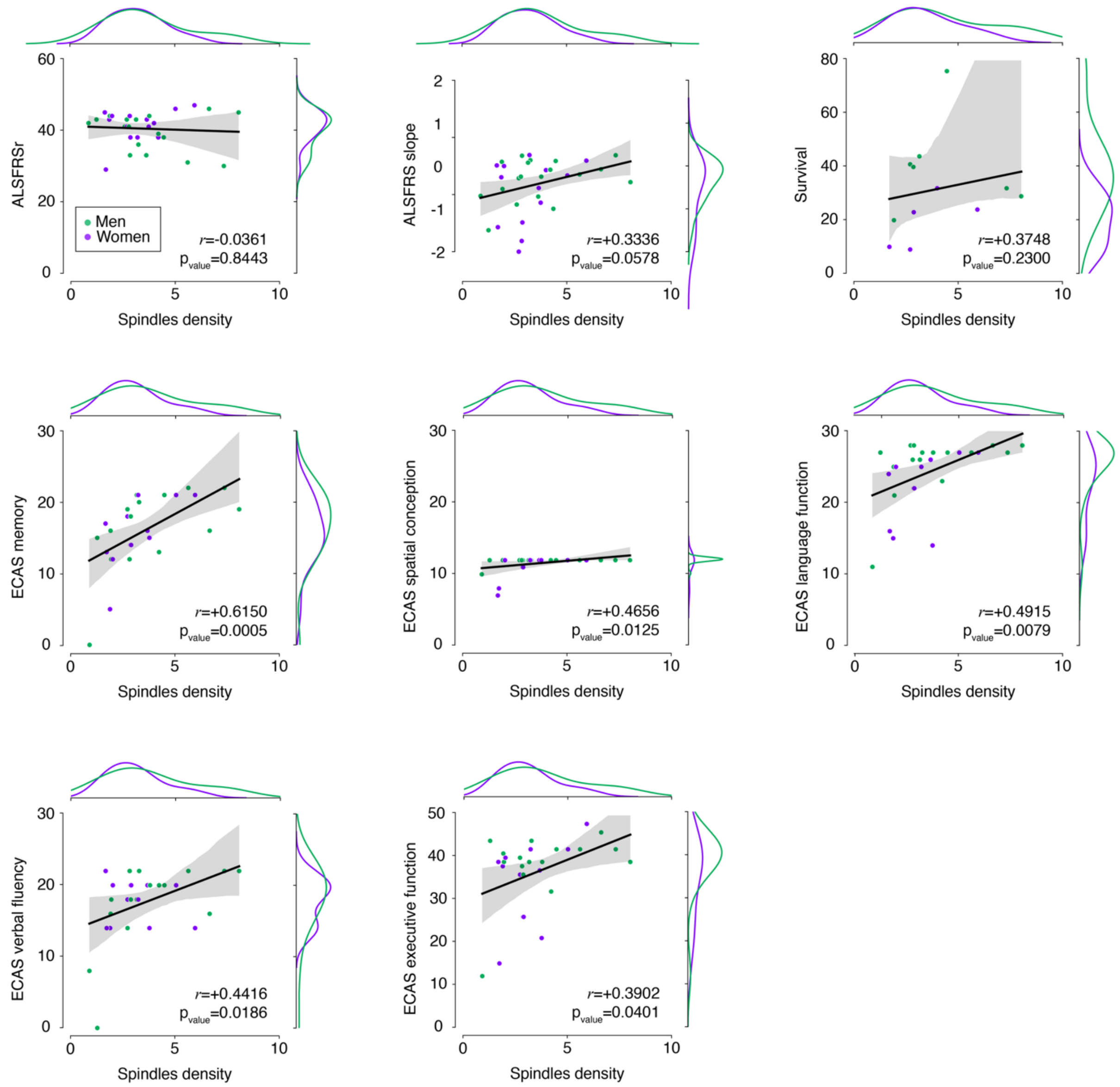

Supplementary Figure 5: Correlation analysis between sleep spindle density and motor and cognitive function in ALS patients.

Correlation between sleep spindle density and indicated parameters.

In all panels, men are shown in green and women in purple.

Spearman p-value was adjusted with FDR-BKY correction. Spearman correlation coefficient *r* and corrected pvalue are indicated. Side distribution represents sex distribution across both variables (men in green, women in purple).

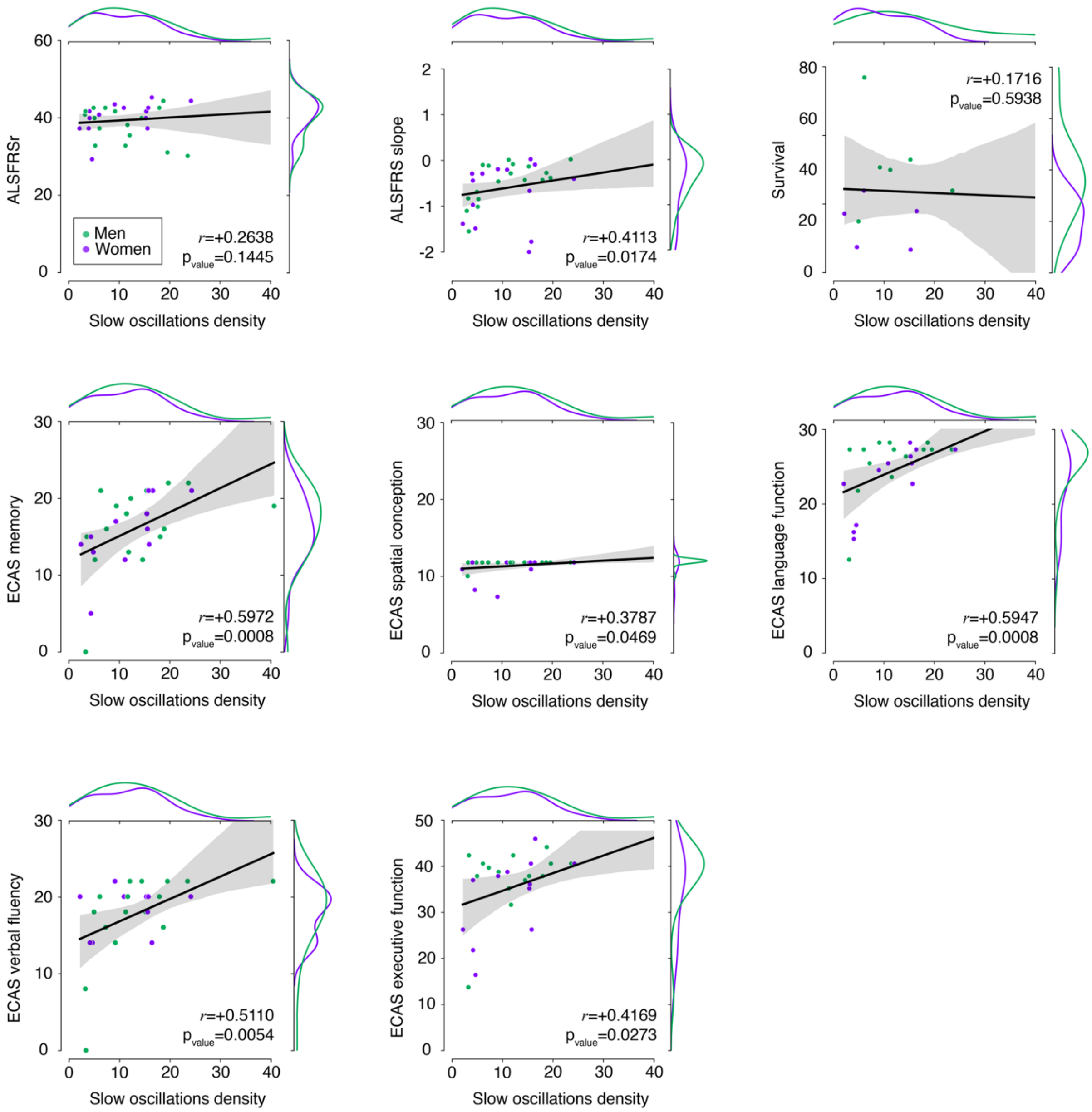

Supplementary Figure 6: Correlation analysis between slow oscillation density and motor and cognitive function in ALS patients.

Correlation between slow oscillation density and indicated parameters.

In all panels, men are shown in green and women in purple.

Spearman p-value was adjusted with FDR-BKY correction. Spearman correlation coefficient *r* and corrected pvalue are indicated. Side distribution represents sex distribution across both variables (men in green, women in purple).

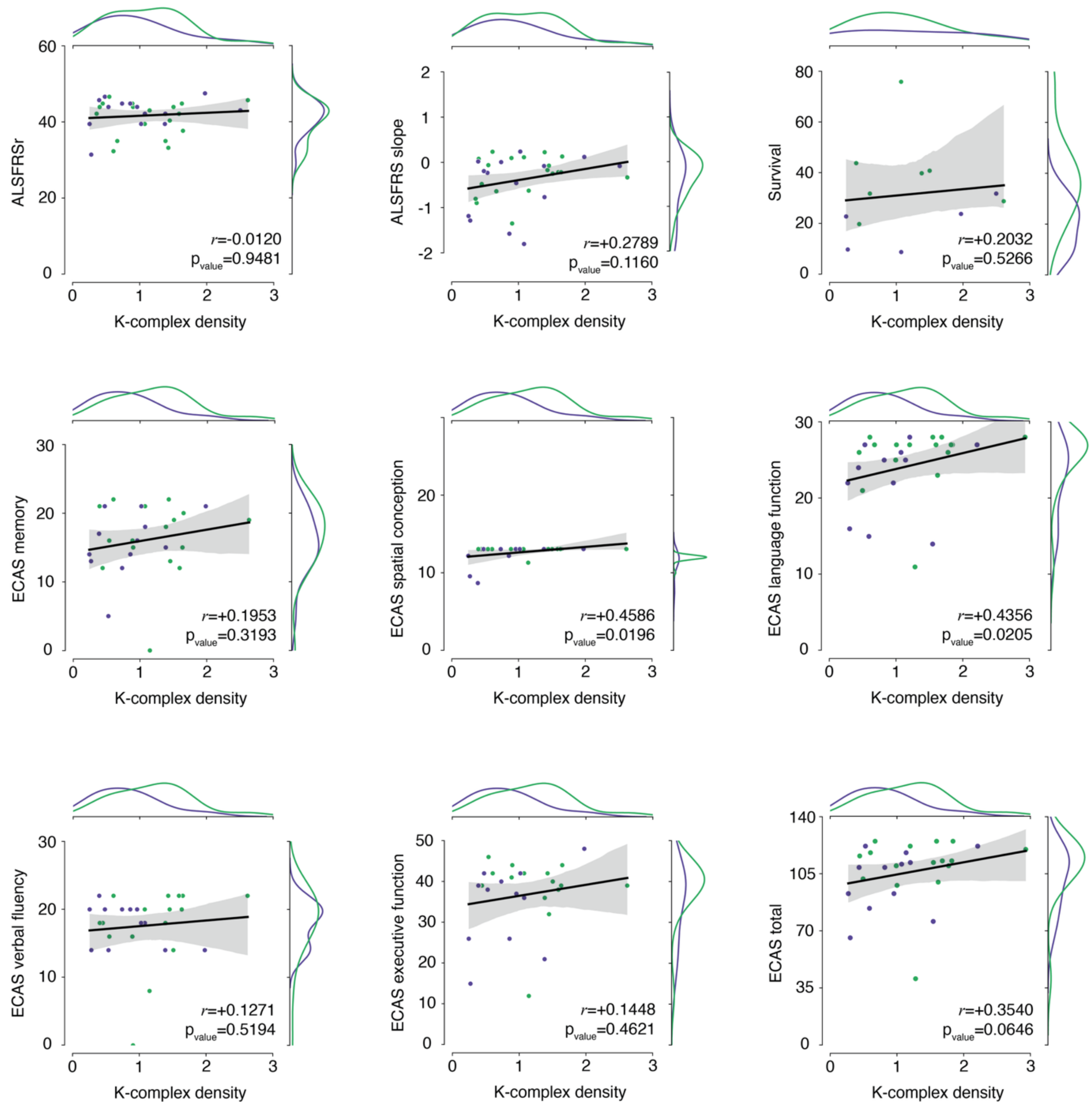

Supplementary Figure 7: Correlation analysis between K-complex density and motor and cognitive function in ALS patients.

Correlation between sleep spindle density and indicated parameters.

In all panels, men are shown in green and women in purple.

Spearman p-value was adjusted with FDR-BKY correction. Spearman correlation coefficient *r* and corrected pvalue are indicated. Side distribution represents sex distribution across both variables (men in green, women in purple).

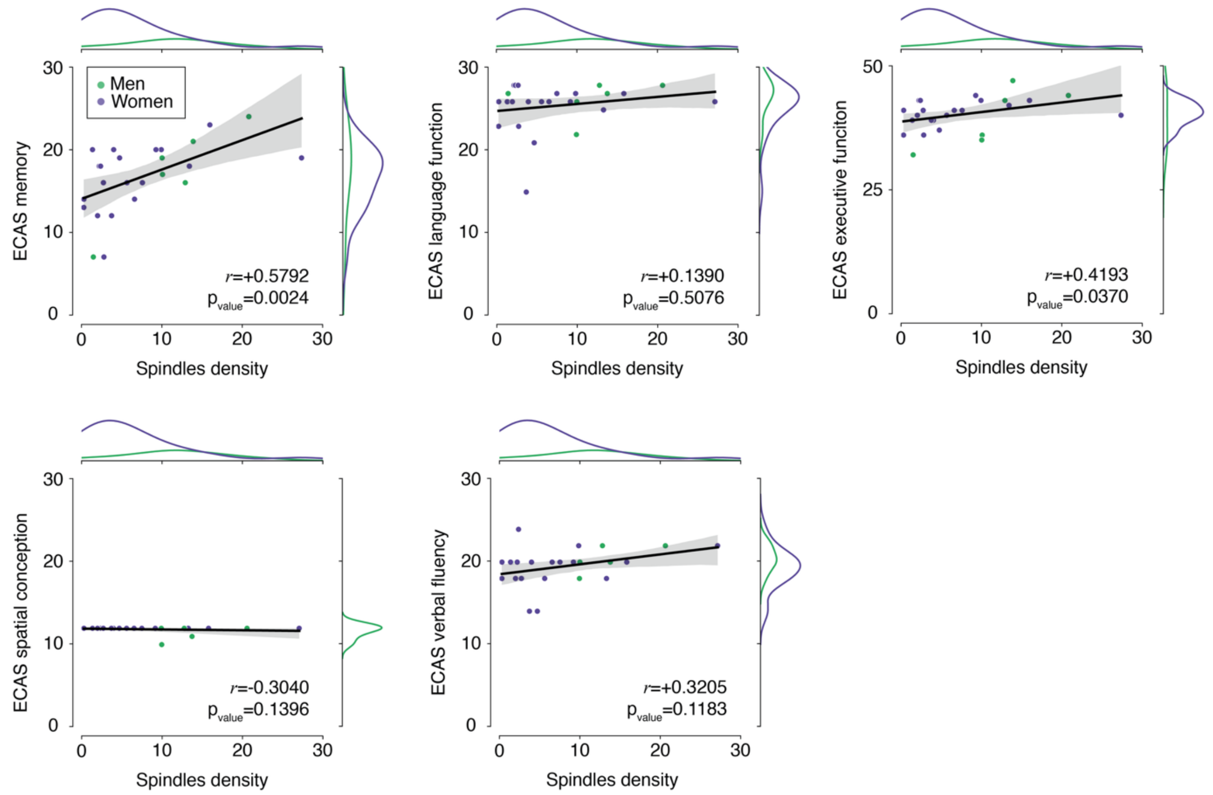

Supplementary Figure 8: Correlation analysis between sleep spindle density and cognitive function in presymptomatic ALS gene carriers.

Correlation between sleep spindle density and indicated parameters.

In all panels, men are shown in green and women in purple.

Spearman pvalue was adjusted with FDR-BKY correction. Spearman correlation coefficient *r* and corrected pvalue are indicated. Side distribution represents sex distribution across both variables (men in green, women in purple).

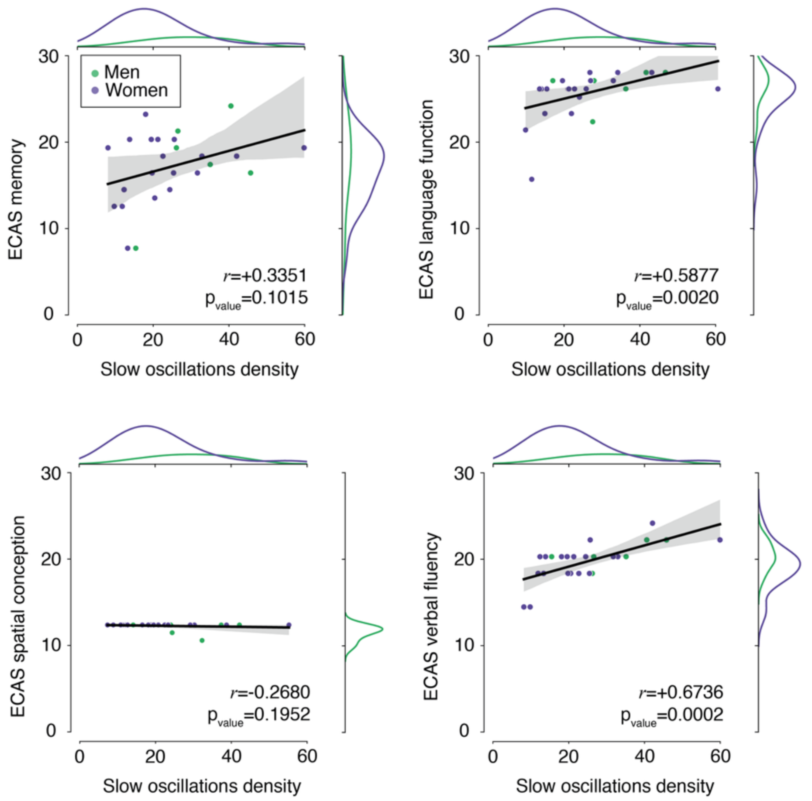

Supplementary Figure 9: Correlation analysis between slow oscillation density and cognitive function in presymptomatic ALS gene carriers.

Correlation between slow oscillation density and indicated parameters.

In all panels, men are shown in green and women in purple.

Spearman pvalue was adjusted with FDR-BKY correction. Spearman correlation coefficient *r* and corrected pvalue are indicated. Side distribution represents sex distribution across both variables (men in green, women in purple).

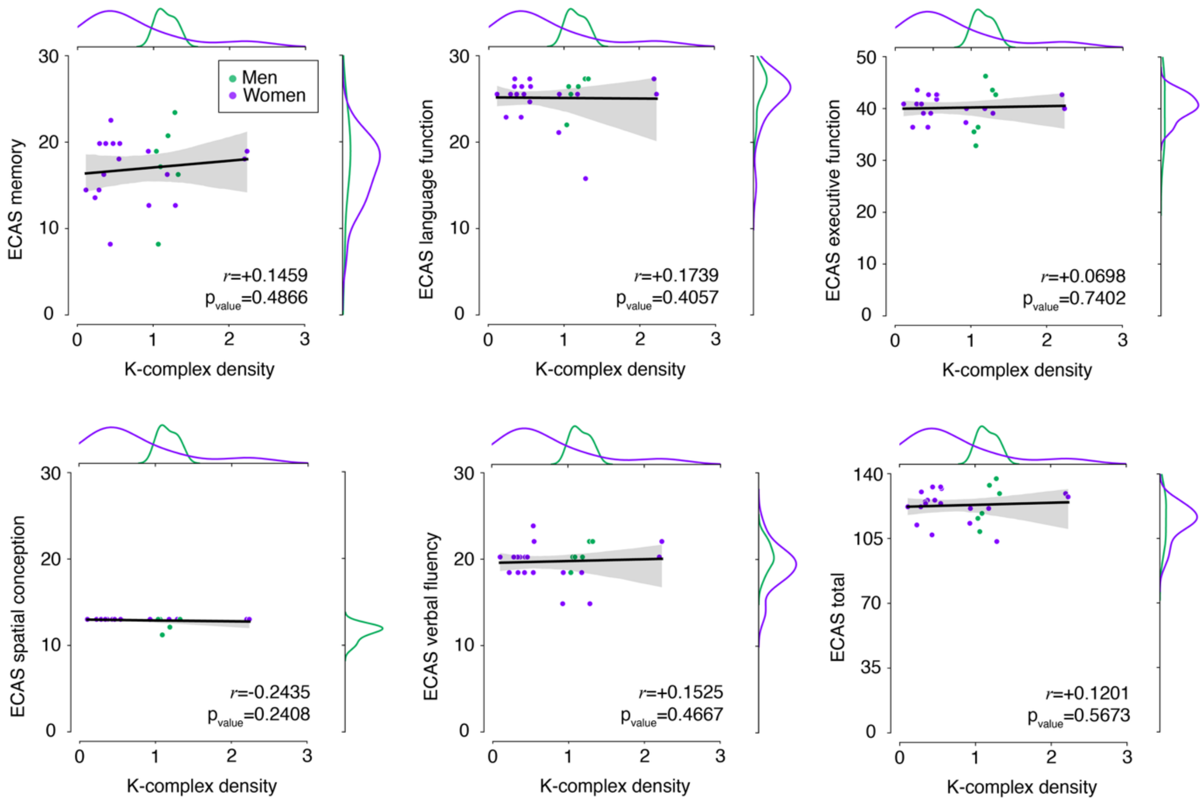

Supplementary Figure 10: Correlation analysis between K-complex density and cognitive function in presymptomatic ALS gene carriers.

Correlation between K-complex density and indicated parameters.

In all panels, men are shown in green and women in purple.

Spearman pvalue was adjusted with FDR-BKY correction. Spearman correlation coefficient *r* and corrected pvalue are indicated. Side distribution represents sex distribution across both variables (men in green, women in purple).

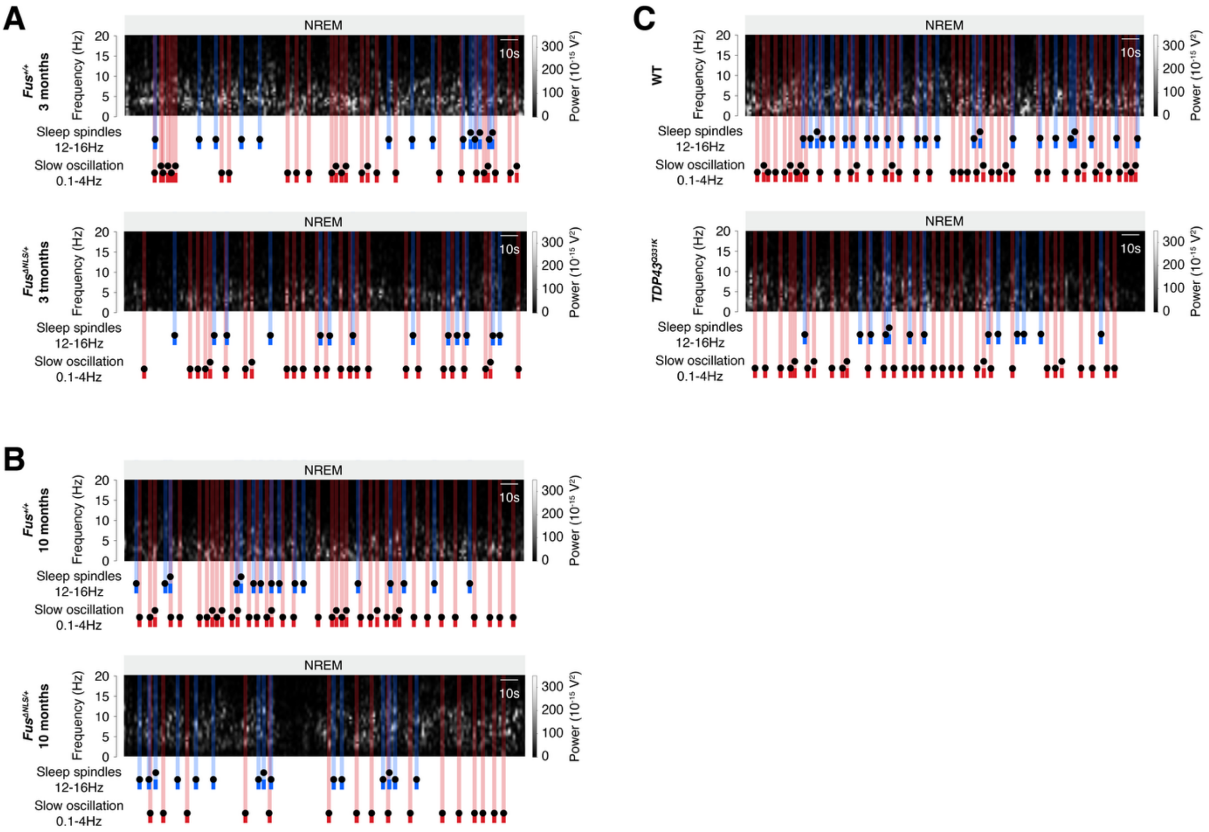

Supplementary Figure 11: Sleep microarchitecture alterations in *Fus*$^{\Delta NLS/+}$ and *TDP-43*^{Q331K} mice.

(**A-C**) Representative spectrogram of mice *Fus*$^{\Delta NLS/+}$ mice and their WT littermates (*Fus*$^{+/+}$) at 3 months of age (**A,** prior to motor symptom onset) or at 10 months of age (**B**) and in *TDP-43*^{Q331K} mice at 10 months of age (**C**).

Sleep spindles are labelled in blue and slow oscillation in red on the spectrogram.

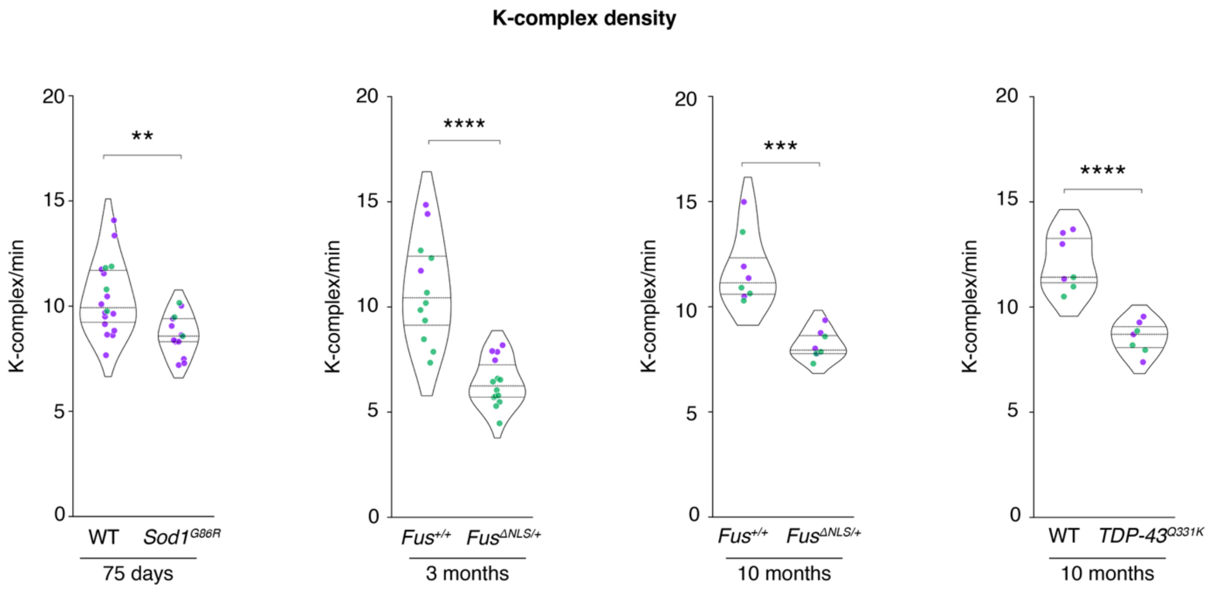

Supplementary Figure 12: additional sleep microarchitecture alterations in *Sod1^{G86R}*, *Fus$^{\Delta NLS/+}$* and *TDP-43^{Q331K}* mice.

Quantification of K-complex density in *Sod1^{G86R}* mice and their non-transgenic WT littermates at 75 days of age, in *Fus$^{\Delta NLS/+}$* mice and their WT littermates (*Fus$^{+/+}$*) at 3 months of age (prior to motor symptom onset) or at 10 months of age and in *TDP-43^{Q331K}* mice at 10 months of age. **** adj. $p_{value}<0.0001$, independent Student's t-test with Welch's t-test with FRD-BKY correction. Data are presented as medians and interquartile ranges. Corrected p_{value} are shown.

Sex effect *Sod1*^{G86R} adj. $p_{value}=0.2282$, *Fus*$^{\Delta NLS/+}$ 3 months adj. $p_{value}=0.0053$, *Fus*$^{\Delta NLS/+}$ 10 months adj. $p_{value}=0.3758$, *TDP-43^{Q331K}* 10 months adj. $p_{value}=0.2943$.

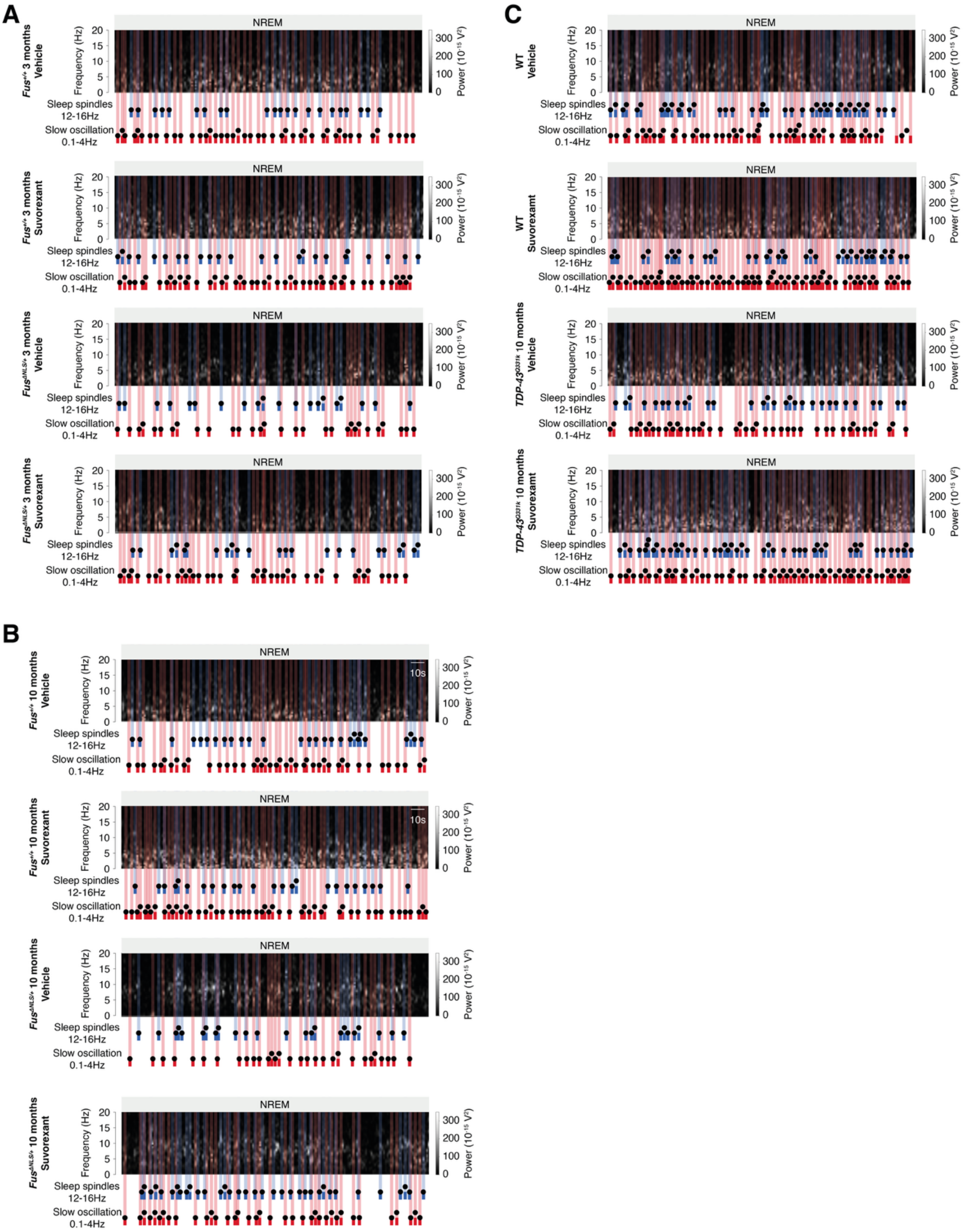

Supplementary Figure 13: Sleep microarchitecture alterations in $Fus^{\Delta NLS/}$ and $TDP\text{-}43^{Q331K}$ mice treated with suvorexant.

(**A-C**) Representative spectrogram of mice administered with either vehicle or Suvorexant. Representative spectrograms for indicated genotypes are shown: $Fus^{\Delta NLS/+}$ mice and their WT littermates ($Fus^{+/+}$) at 3 months of age (**A,** prior to motor symptom onset) or at 10 months of age (**B**) and in TDP-43^{Q331K} mice at 10 months of age (**C**).

Sleep spindles are labelled in blue and slow oscillation in red on the spectrogram.

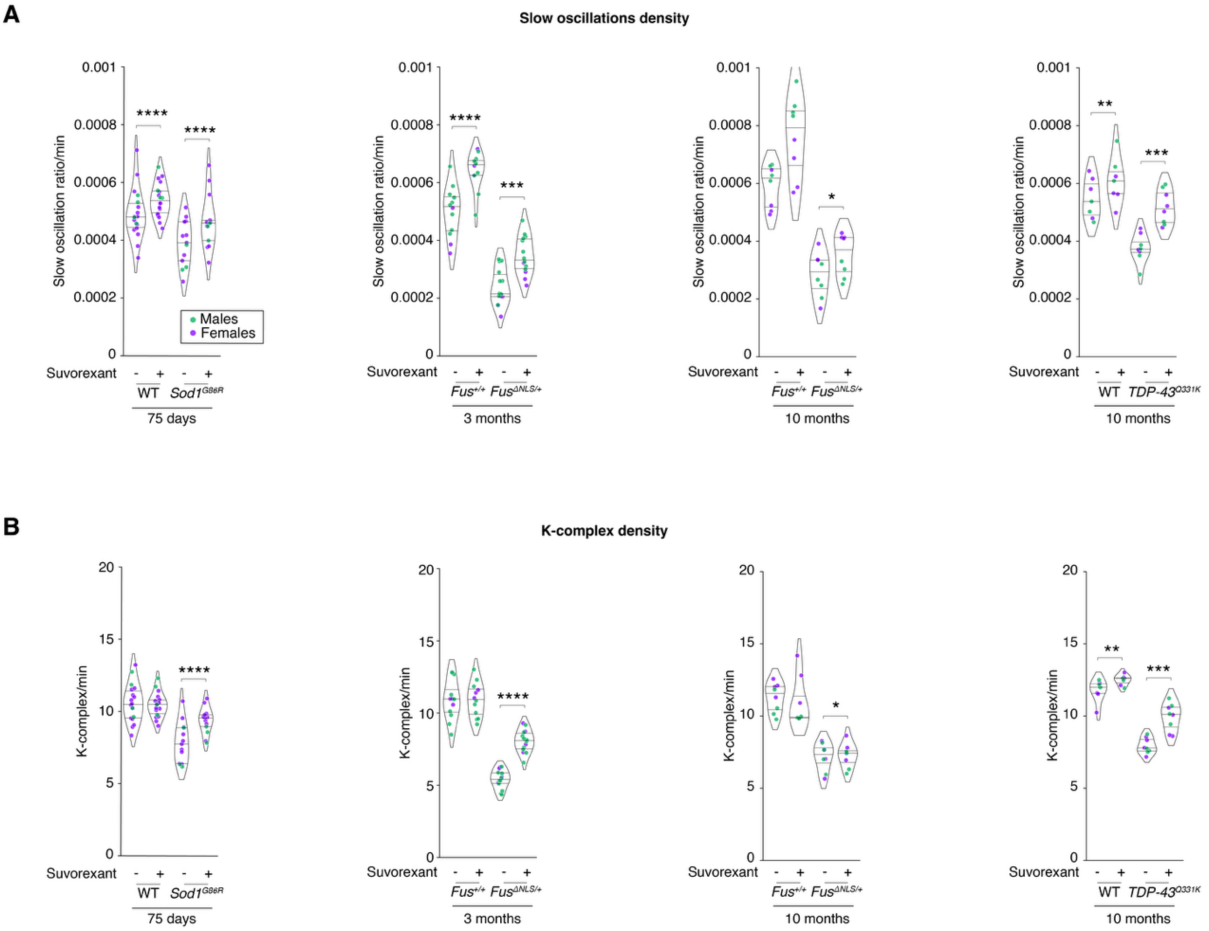

Supplementary Figure 14: additional results on sleep microarchitecture in *Sod1^{G86R}*, *Fus$^{ΔNLS/+}$* and *TDP-43^{Q331K}* mice treated with suvorexant.

(**A-B**) Quantification of slow oscillation density (**A**) and K-complex density (**B**) in *Sod1^{G86R}* mice and their non-transgenic WT littermates at 75 days of age, in *Fus$^{ΔNLS/+}$* mice and their WT littermates (*Fus$^{+/+}$*) at 3 months of age (prior to motor symptom onset) or at 10 months of age and in TDP-43^{Q331K} mice at 10 months of age. Mice were either administered vehicle or suvorexant as indicated.

**** adj. p_{value}<0.0001, Two-Way ANOVA with Dunn's test and FDR-BKY correction. Data are presented as median and interquartile ranges. Corrected p_{value} are shown.

Slow oscillations density: Genotype effect *Sod1*^{G86R} adj. p_{value}<0.0001, *Fus$^{ΔNLS/+}$* 3 months adj. p_{value}<0.0001, *Fus$^{ΔNLS/+}$* 10 months adj. p_{value}<0.0001, *TDP-43*^{Q331K} 10 months adj. p_{value}<0.0001; sex effect *Sod1*^{G86R} adj. p_{value}=0.3006, *Fus$^{ΔNLS/+}$* 3 months adj. p_{value}=0.0167, *Fus$^{ΔNLS/+}$* 10 months adj. p_{value}=0.2069, *TDP-43*^{Q331K} 10 months adj. p_{value}=0.4068.

K-complex density: Genotype effect *Sod1*^{G86R} adj. p_{value}<0.0001, *Fus$^{ΔNLS/+}$* 3 months adj. p_{value}<0.0001, *Fus$^{ΔNLS/+}$* 10 months adj. p_{value}<0.0001, *TDP-43*^{Q331K} 10 months adj. p_{value}<0.0001; sex effect *Sod1*^{G86R} adj. p_{value}=0.1287, *Fus$^{ΔNLS/+}$* 3 months adj. p_{value}=0.2637, *Fus$^{ΔNLS/+}$* 10 months adj. p_{value}=0.5528, *TDP-43*^{Q331K} 10 months adj. p_{value}=0.1215.

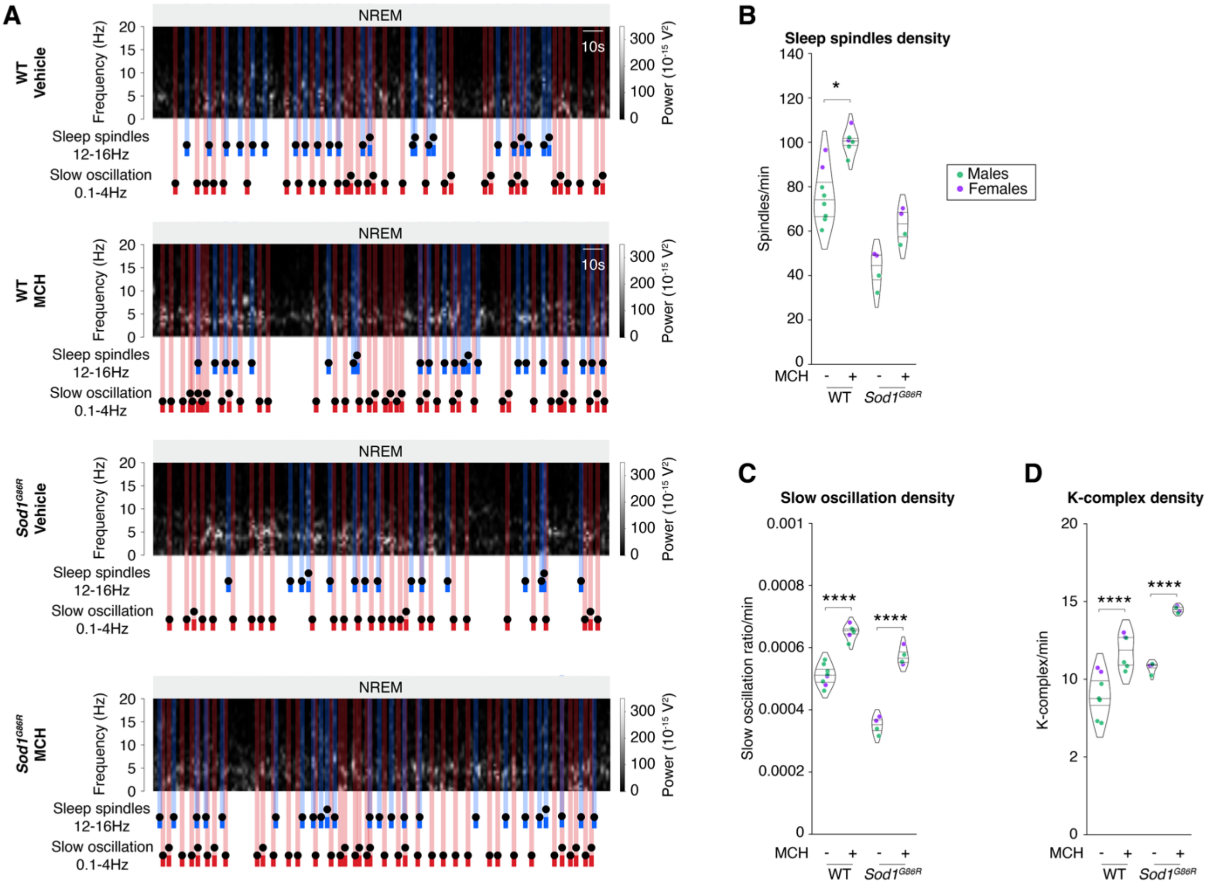

Supplementary Figure 15: Improved sleep microarchitecture by MCH chronic delivery in *Sod1^{G86R}* mice.

(**A**) Representative spectrogram of *Sod1^{G86R}* mice and their non-transgenic wild-type (WT) littermates at 75 days of age (prior to motor symptom onset) administered with either vehicle or MCH via icv cannulation and osmotic minipump delivery. Sleep spindles are labelled in blue and slow oscillation in red on the spectrogram.

(**B-D**) Quantification of sleep spindle density (**B**), slow oscillation density (**C**) and K-complex density (**D**) in *Sod1^{G86R}* mice and their non-transgenic WT littermates treated with either vehicle or MCH at 75 days of age.

*** adj. $p_{value}<0.001$, Two-Way ANOVA with Dunn's test and FDR-BKY correction. Data are presented as medians and interquartile ranges. Corrected p_{value} are shown.

Sleep spindles density: Genotype effect *Sod1*^{G86R} adj. $p_{value}<0.0001$; sex effect *Sod1*^{G86R} adj. $p_{value}=0.3497$.

Slow oscillations density: Genotype effect *Sod1*^{G86R} adj. $p_{value}<0.0001$; sex effect *Sod1*^{G86R} adj. $p_{value}=0.2409$.

K-complex density: Genotype effect *Sod1*^{G86R} adj. $p_{value}<0.0001$; sex effect *Sod1*^{G86R} adj. $p_{value}=0.6290$.

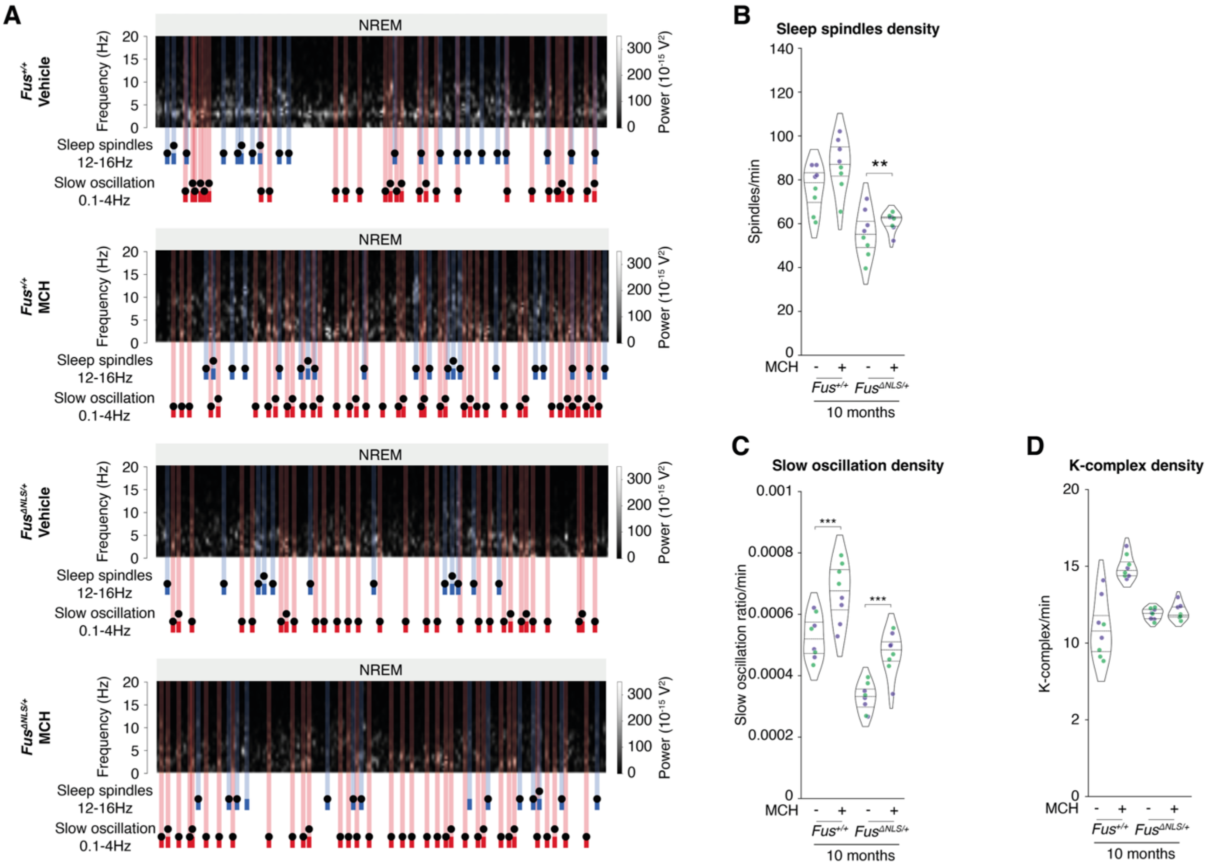

Supplementary Figure 16: Improved sleep microarchitecture by MCH chronic delivery in *Fus*$^{\Delta NLS/+}$ mice.

(A**)** Representative spectrogram of *Fus*$^{\Delta NLS/+}$ mice and their non-transgenic wild-type (WT) littermates at 75 days of age (prior to motor symptom onset) administered with either vehicle or MCH via icv cannulation and osmotic minipump delivery. Sleep spindles are labelled in blue and slow oscillation in red on the spectrogram.

(**B-D**) Quantification of sleep spindle density (**B**), slow oscillation density (**C**) and K-complex density (**D**) in *Fus*$^{\Delta NLS/+}$ mice and their WT littermates (*Fus*$^{+/+}$) mice treated with either vehicle or MCH at 10 months of age.

*** adj. p_{value}<0.001, Two-Way ANOVA with Dunn's test and FDR-BKY correction. Data are presented as median and interquartile ranges. Corrected p_{value} are shown.

Sleep spindles density: Genotype effect *Fus*$^{\Delta NLS/+}$ 10 months adj. p_{value}<0.0001; sex effect *Fus*$^{\Delta NLS/+}$ 10 months adj. p_{value}=0.1408.

Slow oscillations density: Genotype effect *Fus*$^{\Delta NLS/+}$ 10 months adj. p_{value}<0.0001; sex effect *Fus*$^{\Delta NLS/+}$ 10 months adj. p_{value}=0.6316.

K-complex density: Genotype effect *Fus*$^{\Delta NLS/+}$ 10 months adj. p_{value}<0.0001; sex effect *Fus*$^{\Delta NLS/+}$ 10 months adj. p_{value}=0.1903.

Supplementary Tables

Supplementary Table 1: Correlation between sleep microarchitecture and clinical parameters in ALS patients

Spearman r and adjusted p-values are indicated for each comparison.

	Density of sleep spindles		Density of slow oscillations		Density of K-complexes	
	r	p_{value}	*r*	p_{value}	*r*	p_{value}
**ALSFRSr**	-0,0361	0,8443	0,2638	0,1445	0,0120	0,9481
**ALSFRS Slope**	0,3336	0,0578	0,4113	0,01741	0,2789	0,1160
**Survival**	0,3748	0,2300	0,1716	0,5938	0,2032	0,5266
**ECAS Memory**	0,6150	0,0005	0,5972	0,0008	0,1953	0,3193
**ECAS Spatial Conception**	0,4656	0,0125	0,3787	0,0469	0,4586	0,0196
**ECAS Language function**	0,4915	0,0079	0,5947	0,0009	0,4356	0,0205
**ECAS Verbal fluency**	0,4416	0,0186	0,5110	0,0054	0,1271	0,5194
**ECAS Executive function**	0,3902	0,0400	0,4169	0,0273	0,1449	0,4621
**ECAS Total**	0,7178	0,000017	0,7410	0,0000065	0,3540	0,0646

Supplementary Table 2: Correlation between sleep microarchitecture and clinical parameters in presymptomatic gene carriers

Spearman *r* and adjusted p_{values} are indicated for each comparison.

	Density of sleep spindles		Density of slow oscillations		Density of K-complexes	
	r	p_{value}	*r*	p_{value}	*r*	p_{value}
**ECAS Memory**	0,5792	0,0024	0,3351	0,1015	0,1459	0,4866
**ECAS Spatial Conception**	-0,3040	0,1396	-0,2680	0,1952	-0,2435	0,2408
**ECAS Language function**	0,1390	0,5076	0,5877	0,0020	0,17393	0,4057
**ECAS Verbal fluency**	0,3205	0,1183	0,6736	0,00022	0,1525	0,4667
**ECAS Executive function**	0,4193	0,0370	0,4406	0,0275	0,0698	0,7402
**ECAS Total**	0,4748	0,0165	0,5764	0,00256	0,1201	0,5673
